# Osteopontin in pancreatic cancer: A systematic review

**DOI:** 10.3892/mi.2026.310

**Published:** 2026-03-23

**Authors:** Georg F. Weber

**Affiliations:** James L. Winkle College of Pharmacy and Cancer Center, University of Cincinnati Cancer Center, Cincinnati, OH 45267, USA

**Keywords:** pancreatic cancer, osteopontin, tumor progression, biomarker, tumor immunology, premalignant lesion, smoking, pancreatitis, diabetes

## Abstract

The present study analyzed the available literature on osteopontin in pancreatic cancers. Before the cut-off date, PubMed listed 105 pertinent references, plus 39 results covering osteopontin in non-cancerous conditions of the pancreas. The molecule fulfills physiologic roles in pancreatic development and function, including islet survival and protection from hyperglycemia. Osteopontin has been found upregulated in cancers of the pancreas and may serve as a biomarker for transformation, progression or survival prospects, particularly in conjunction with other molecular indicators. It has been reliably corroborated as a blood biomarker for these malignancies. In particular, the measurement of the cancer-specific osteopontin splice variants OPN-b and OPN-c achieves upgraded diagnosis. Animal and cellular models have elucidated the functions of osteopontin in support of the metastasis, tropism and stemness of the cancer cells, as well as roles in angiogenesis and chemoresistance. As the full-length form of osteopontin, OPN-a, serves as an inducer cytokine for cellular immunity, it has been characterized by several studies as a regulator in pancreatic tumor immunology, particularly in macrophages. Osteopontin induction and biologic effects are associated with various premalignant and predisposing conditions, including intraductal papillary mucinous neoplasms, smoking, pancreatitis and kidney disease. In diabetes and obesity, the molecule plays complex roles that may either attenuate or promote disease progression. While osteopontin has emerged as a key physiological regulator of pancreatic functions, its aberrant expression and splicing in cancers of the pancreas supports tumor progression and may serve early detection as well as prognostication. The splice variants have potential to become therapeutic targets in anti-metastasis regimens.

## Introduction

Cancer of the pancreas is often detected at a late stage. At that juncture, it has commonly disseminated and is therefore afflicted by a poor prognosis. A better understanding of the molecular mechanisms underlying the disease, in particular its progression and dissemination, should point the way to improved clinical care. In tumor stage progression and metastasis formation of this malignancy, osteopontin (also known as OPN or SPP1) has been known to be a major contributor (1-3). Hence, it is of importance to elucidate the knowledge base about this secreted phosphoglycoprotein, pertaining to the induction of its aberrant expression and its splice variants (4,5) in pancreatic cancers. Such insights may improve early detection/diagnosis and can point the way toward novel therapeutic targets associated with tumor spread. For this purpose, the present systematic review was conducted, analyzing studies found on PubMed-listed database on this subject (Fig. S1).

## Data and methods

### Source literature

Publications in the PubMed database with the key phrase ‘pancreas cancer OR pancreatic cancer AND osteopontin’ yielded 105 references (cut-off date, December 31, 2025). A total of 26 articles were found not to be pertinent, predominantly as they studied cancers of different organ origin (mostly liver cancers, but also colorectal and gastric cancers); 1 article had been retracted. Of note, ‘osteopontin AND pancreas NOT cancer’ produced 39 results (cut-off date, December 31, 2025). This search captured papers on the roles of osteopontin in development and organ physiology, as well as in various predisposing conditions. In total, six hits were found not to be pertinent to pancreatic function or to osteopontin (Fig. S2).

### Analysis

The mined articles were downloaded, summarized and organized according to the aspect covered in the original studies. They include the history and molecular characteristics of osteopontin, pancreas development and function (development, expression, function), tumor tissue (expression, marker combination, tumor progression, survival), tumor blood (protein biomarker, RNA biomarker, marker combination, progression, post-treatment recovery), tumor immunology, tumor models (metastasis, tropism, stemness, angiogenesis, chemoresistance), predisposition and early lesions (prediagnosis and premalignancy, smoking, pancreatitis, kidney disease), and diabetes and obesity (prediabetes, type 1 diabetes, type 2 diabetes, cancer connection). Multiple references are relevant to several subsections of the present systematic review and are therefore cited accordingly.

### Data extraction

All reviewed publications were tabulated in the chronological order of their publication. In a separate table, patient-derived results were organized with more detail. They are arranged according to the cancer feature (or other disease feature) addressed. Signal transduction connections are depicted in the figures.

## Results

### History and molecular characteristics of osteopontin

Studying various mammalian tumor cell lines in 1979, Senger *et al* (6,7) described transformation-specific secreted phosphoproteins, including one at a size of 58 kDa. The cloning of osteopontin (also known as OPN, secreted phosphoprotein 1, SPP1 or bone sialoprotein) was reported independently in 1986(8). With the protein sequence at hand, the description in 1988/89, by the groups of Denhardt [Craig *et al* (9)] and Senger *et al* (10,11), that the phosphoglycoprotein osteopontin is prominently secreted by cancer cells, has prompted much ensuing research into the roles played by this molecule in tumor progression (to date, ~3,040 publications on ‘osteopontin AND cancer’ in PubMed). Mechanistic insight was gained by the identification of the receptor integrin α_V_β_3_ (12) and the discovery that a metastasis-associated splice variant of CD44 also serves as a receptor (13). Additional integrins have been identified as osteopontin binding partners. A new angle arose in 2006, with the report that osteopontin splice variants are selectively produced by malignant cells, but not by untransformed cells (14). It has impacted the study of osteopontin in pancreatic and other types of cancer.

The expression and distribution of osteopontin in the luminal epithelial surfaces of tissues, including the pancreas, was published in 1992(15). Initial cursory mention of this protein in pancreatic cancer dates back to a 1997 review on neoplastic cell adhesion to CD44(16). Since then, a literature has evolved that analyzes osteopontin and its roles in cancers and other conditions afflicting the pancreas (Table SI).

**Figure 1:**
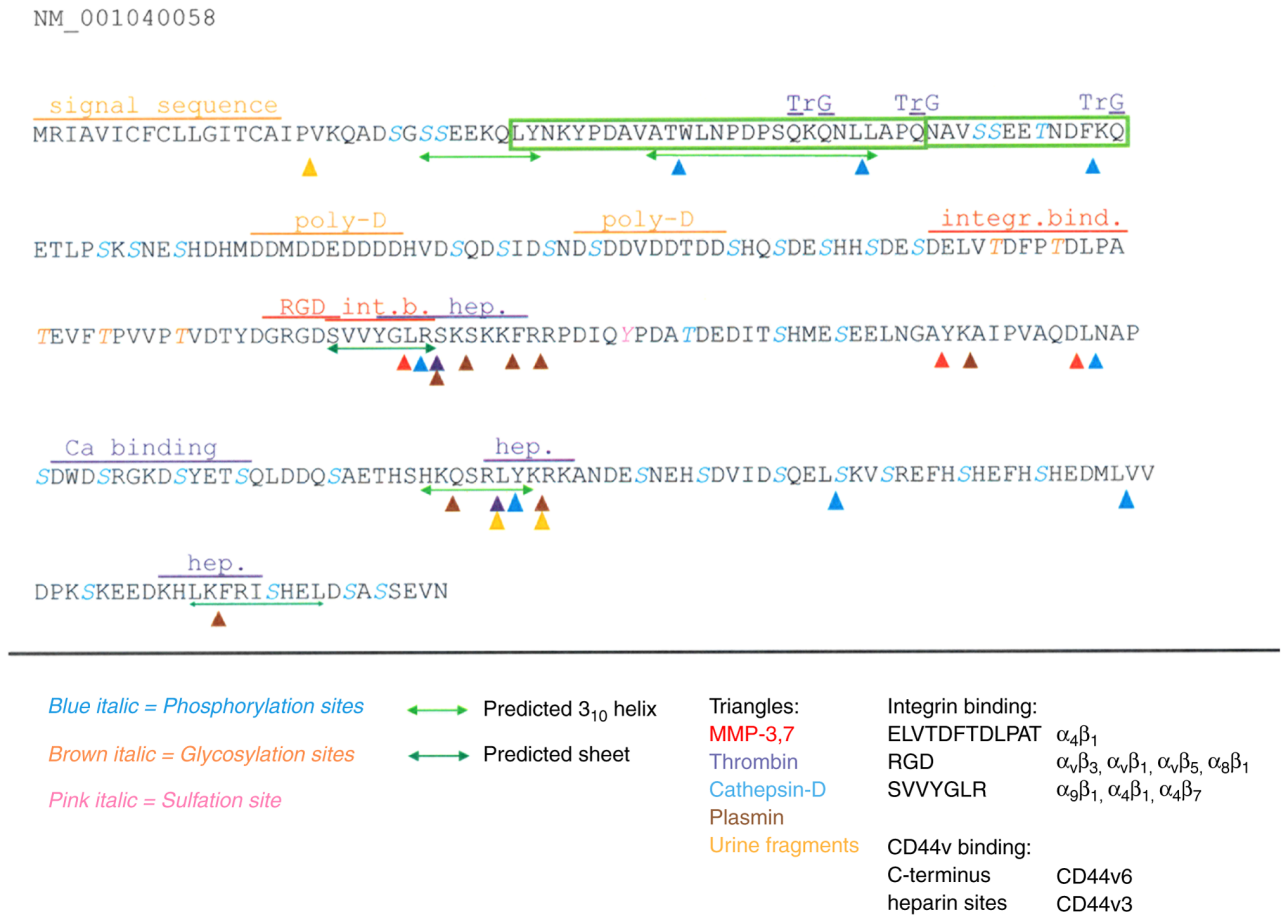
Osteopontin. The image displays the osteopontin sequence with key structural characteristics highlighted by color coding. The domains (exons) spliced out in OPN-b and OPN-c are contained in green boxes. Functional sites are identified above the protein sequence. Susceptibility to proteases is shown in color-coded triangles below the sequence. The protein is believed to be largely unstructured; the few tentatively identified helices or sheets are marked by green double-arrows below the sequence.

The osteopontin protein (Fig. 1) is characterized as very acidic, largely unstructured, highly phosphorylated and glycosylated, as well as possessing abundant calcium-binding capacity. Osteopontin can engage several cell surface receptors, prominently integrins and CD44v (a splice variant of CD44, entailing variant exons 3-6). Thrombin cleavage (10) separates the integrin-engaging N-terminus from the heparin- and CD44v-ligating C-terminus (17). There are two heparin binding sites (18) likely involved in the interaction with CD44v, but also modulating the unfolding of a core element in the protein (19,20). In the bone, osteopontin attaches to hydroxyapatite (21). Furthermore, the protein has been reported to harbor an interaction domain with factor H (22), a site for engagement of neuropilin-1(23), and a domain to bind to inducible t-cell costimulator ligand (ICOSL) (24). Whereas the physiologic role of osteopontin lies in tissue remodeling and immune activation (systemically), as well as the regulation of calcification (in bone, breast and kidneys) (25-29), in cancer, it mediates the survival of deadherent cells (30), as well as migration and invasion (17) during the process of tumor dissemination.

Humans generate multiple gene products of osteopontin through alternative splicing (14,31) or through an alternative transcription start site (32). With the exception of the alternative transcriptional start site (which produces an intracellular form), all osteopontin variants are secreted. Tumor progression genes are aberrantly expressed or spliced in malignancies (4,5), and osteopontin overexpression and/or splicing has been associated with a number of aggressive tumors (33-35). While alternative splicing of the osteopontin mRNA is formally possible in several species and cell types, it has only been reliably shown to arise in human transformed cells (36). Specifically, the role for the metastasis-promoting variant OPN-c in cancer progression has been elucidated as a gain-of-function at the splice junction that ligates SLC7A11, activates peroxides, and facilitates mitochondrial biogenesis (30).

Osteopontin is encoded by the spp1 gene, located on chromosome 4 in locus 4q13.22, which has 7 exons, the first of which is silent. The gene belongs to the SIBLING family (37), which stands for ‘small integrin binding ligand N-linked glycoproteins’ (38).

### Pancreas development and function. Development

Matricellular proteins mediate tissue morphogenesis and homeostasis by modulating cell-matrix and cell-cell interactions. Osteopontin is a marker of undifferentiated pancreatic precursors and ductal tissues. The specific, dynamic profile of its expression in embryonic pancreatic tissues reflects its participation in processes involving cell migration or cell-cell interactions (39). In the maintenance of duct cell identity, multiple cellular subpopulations retain progenitor capacity. An epithelial-mesenchymal transitory axis may arise in three duct subpopulations. Osteopontin marks a cell type enriched in progenitor capacity. It serves a regulator of the epithelial-mesenchymal transitory fate decision, and it is required for differentiation past progenitor function and for maintenance of duct function as reflected in carbonic anhydrase activity. In its presence, the levels of markers of mature duct cells, including HNF1B, SOX9 and KRT19, are increased. In its absence, there is an increase in markers associated with epithelial-mesenchymal transition, including VIM, ZEB1, TWIST1 and MMP2(40). However, osteopontin-deficient pancreata have not exhibited obvious alterations in the morphology or differentiation of these tissues (39).

Selenium forms part of glutathione peroxidase, which assumes complex roles in metabolic syndrome. Offspring with metabolic syndrome had a lower body weight, with males having a lower body mass index and growth indicators in serum. Females, in particular, had lower levels of serum insulin. All offspring presented a repletion of selenium in pancreas, kidneys, and thyroid as well as depletion in heart and muscle. Serum osteopontin was not altered by either sex or high-fructose diet (41).

*Expression*. In healthy or tumor-surrounding untransformed pancreatic tissue, osteopontin immunoreactivity has been variably described. An *in situ* hybridization analysis did not find detectable expression in healthy tissue (42); this was also the case by histology in normal pancreatic glandular epithelia (43). Healthy pancreatic tissue samples displayed a range from no staining (20%), via moderate staining, to high staining (~10%) (44). In another study, however, almost 60% of the healthy controls were reported to express osteopontin (45).

Immunohistochemical staining for osteopontin was positive for the epithelium lining in large and small pancreatic ducts (15,46) or localized in ductal connective tissue (47). A diffuse cytoplasmic staining pattern according to immunohistochemistry was present in 5% of the ductal cells, with either occasional expression in islets (48) or expression in all islet cells (46). Osteopontin is localized, to a varying degree, in connective tissue around acini (47). A diffuse cytoplasmic staining pattern according to immunohistochemistry was present in 30% of the acinar cells (48). By immunofluorescence, the central areas of acini were exclusively stained (49). No cells demonstrated nuclear staining (44).

The discrepancies in the reported immunohistochemistry staining may be a consequence of the rich variations in posttranslational modifications of osteopontin (including phosphorylation, glycosylation, multiple sites for proteolytic cleavage, binding to calcium, heparin, or hyaluronate). Due to these structural adaptations, the choice of the antibody to be utilized for the staining can have a major impact on the results [as was previously published for ELISA applications (50)]. Since the osteopontin field has grown (currently almost 13,800 publications on this molecule in PubMed), so has the number of commercially available antibodies (~1,120 antibodies to the human protein in Biocompare). Thorough standardizations would be a requirement for diagnostic pathology usage, but have not been conducted yet.

*Function*. In β-cells, osteopontin is protective against both cytotoxicity and hyperglycemia (51-53). Insulin secretion is impaired with increasing age, suggesting that aging induces specific transcriptional changes in human islets. A total of 346 genes that co-vary with age included increased transcription of genes linked to senescence and downregulation in several aspects of the cell cycle machinery. Also correlating with age is an upregulation in osteopontin expression (51).

The maintenance of osteopontin expression in the pancreatic tissues of adults argues for a function of this protein in injury and pathologic responses (39). Pancreatic α-cells produce osteopontin, which facilitates insulin release from stressed β-cells. The protective stress response extends to metabolism (52).

i) The incretin hormone glucose-dependent insulinotropic polypeptide (GIP) promotes pancreatic β-cell function by potentiating insulin secretion and β-cell proliferation. GIP stimulated osteopontin mRNA and protein expression, thus exerting effects on β-cell survival in islets (proliferation by insulin-secreting cells, preservation of functional β-cell mass), as well as the regulation of adipocyte metabolism in fat tissue. Osteopontin expression is lower in carriers of the A allele in the receptor GIPR (site rs10423928). The effect is specific for GIP, as GLP-1 has no impact on osteopontin expression, regardless of the glucose concentration (53).

ii) The transmembrane receptor sortilin-related VPS10-domain containing receptor 2 (SORCS2) in the pancreas is predominantly expressed in islet α-cells. Its activity safeguards insulin granule formation and release from glucose-stressed β-cells. The loss of SORCS2 expression coincided with inability of the α-cells to produce osteopontin. Consistently, β-cells in SORCS2-deficient islets exhibited gene expression patterns indicative of aggravated cell stress, exhibited defects in insulin granule maturation, and had a blunted glucose response (52).

### Tumor tissue. Expression

Rather consistently, immunohistochemistry evaluations have found ~70% osteopontin expression in pancreatic cancer (Table SII). In a comparison between undifferentiated carcinoma and ductal adenocarcinoma, both had close to 70% positivity for osteopontin (54). Osteopontin staining was present in 60% (48) or 74% (45) of primary adenocarcinomas. Osteopontin was strongly expressed in 70% of adenocarcinoma tissues, but only in ~30% of surrounding healthy tissue (55). The staining intensity in the tumors displayed a range, being absent in ~35%, weak to moderate in 50% and high in 15% (44). Immunofluorescence analysis of cancer tissue from patients with invasive ductal adenocarcinoma, the majority of whom were smokers, revealed elevated amounts of osteopontin in the malignant ducts and the surrounding pancreatic acini (56).

Stromal osteopontin was present in >90% of cancers, but with large variations in intensity and staining distribution (47). A substantial portion of the surrounding fibroblasts displayed staining (44). Immunofluorescence revealed weak intracellular staining in all cancer cells, while stroma stained positive but even weaker than the cancer cells (49).

Consistent with the secretion of osteopontin via the Golgi organ, its subcellular localization in histochemistry is predominantly cytoplasmic. Where present, benign and malignant pancreatic ductal cells displayed cytoplasmic and luminal staining. The expression in adenocarcinoma cells was located mostly in the cytoplasm (45). A substantial portion of the surrounding fibroblasts in the stroma presented a cytoplasmic staining pattern as well (44).

An analysis of gene expression profiles in Oncomine identified the upregulation of osteopontin in pancreatic cancer as not reaching significance. However, in a separate evaluation of ductal adenocarcinoma and intraepithelial neoplasia over healthy tissue, each neoplasia was associated with significant overexpression (57). Using RT-qPCR, there was a 2.2-fold increase in osteopontin mRNA in adenocarcinoma and a 1.6-fold increase in chronic pancreatitis samples, respectively, compared to healthy pancreatic tissues (48). In patients with adenocarcinoma, osteopontin was expressed in 43% with variable contributions by the splice variants OPN-a, OPN-b and OPN-c (58). The variant form OPN-c was present in 87% of invasive ductal adenocarcinoma lesions, of whom 73% were smokers. The levels of OPN-c correlated well with higher expression levels of total osteopontin in the tissue and serum from these patients (59,60). The splice variants have great marker potential, as osteopontin splice forms were present only in ~15% of tumor-surrounding healthy specimens (58). However, *in situ* hybridization did not find detectable expression in the adenocarcinoma cells (42), which could indicate that the technique may lack sensitivity compared to PCR-based approaches.

Osteopontin is also associated with less common types of pancreatic cancers.

i) Ampullary adenocarcinoma is an aggressive cancer with poor prognosis, which can be difficult to distinguish from ampullary adenoma prior to resection. Osteopontin mRNA is substantially elevated in this cancer. Its measurement may aid in the early detection and differential diagnosis of patients with periampullary lesions (61). By *in situ* hybridization, tissue osteopontin was detectable more strongly in ampullary cancer than in pancreatitis or in healthy tissue (62). Osteopontin expression in the cancer cells was not associated with prognosis; however, the expression of osteopontin and location of tumor-associated macrophages in bulky ampullary cancer predicted recurrence (63).

ii) Osteoclast-like giant cell tumors are rare neoplasms of the pancreas and mostly associated with ductal adenocarcinomas. In the rare case of an osteoclast-like giant cell tumor associated with mucinous cystadenocarcinoma, osteopontin was expressed in the osteoclast-like giant cells but not in the mononuclear tumor cells (64).

*Marker combination*. Since osteopontin had been corroborated as a pancreatic cancer marker, the utility of its association with other markers has been studied (Table SIII).

i) Osteopontin and the transcription factor FOXM1 were significantly upregulated in pancreatic cancer tissues and were linked to poor clinical outcome (65). In tumor tissues, there was no correlation of the RNA level between KRAS and TP53 mutational status and osteopontin expression (58). In undifferentiated cancers and adenocarcinomas, there was no correlation between osteopontin expression and E-cadherin or β-catenin expression (54).

ii) Multiple reports have focused on ductal adenocarcinoma lesions: The osteopontin staining levels correlated with MMP-9 and vascular endothelial growth factor (VEGF) (66), correlated and colocalized with the chemokine CCL-2 (MCP-1) in transformed cells and in the malignant ducts (67), and, together with immunostaining for LIM homeobox transcription factor 1α (LMX1A), were associated with advanced nuclear grades and advanced stages (while both markers were undetectable in healthy pancreatic glandular epithelia) (43). Whereas osteopontin and RAN mRNA levels highly correlated with each other in tumor cells, adjacent non-malignant and benign pancreatic tissues, the levels of either did not correlate with venous lymphatic invasion, diabetes, obesity, T stage, body mass index, or survival (68,69).

iii) Pancreatic cystic lesions may be benign, requiring observation, or cancerous, requiring surgery. Lower osteopontin, sTIE-2 (soluble form of the receptor for angiopoietins) and leptin levels in cystic fluid were associated with cancer (70).

A personalized adenocarcinoma-derived organoid chip with functional endothelial barrier (to simulate the vascular permeation and tumor interactions) was developed for biomarker detection and functional drug sensitivity testing. Tumor-specific biomarkers, including osteopontin, CA-19.9, TIMP-1, macrophage inhibitory cytokine-1 (MIC-1), the adhesion receptor ICAM-1 and soluble AXL (a receptor tyrosine kinase) were consistently present in the chip outflows (71).

*Tumor progression*. Osteopontin staining was present in 60% of primary adenocarcinomas and in 70% of lymph node and liver metastases (48). The small GTPase RAN drives pancreatic cancer metastasis by modulating androgen receptor (AR) expression. In metastatic lymph node tissues, there were elevated levels of RAN, osteopontin and AR (69).

The association of total osteopontin with cancer progression has been very variably reported as being absent, being reflective of good prognosis, or being an indicator of poor outcome. It is conceivable that the inconsistencies were due to the lack of distinction between tumor-derived osteopontin splice variants, which facilitate progression, and osteopontin produced by the host in response to tissue damage, which supports remodeling and healing. The measurement of cancer-associated osteopontin splice forms may be a more accurate tool for prognostication of progression than pan-osteopontin.

i) In premalignant intraductal papillary mucinous neoplasms, osteopontin was a biomarker for the surveillance of carcinogenic progression (72).

ii) Osteopontin expression and the location of tumor-associated macrophages in bulky ampullary cancer predicted recurrence (63). While being undetectable in normal pancreatic glandular epithelia, osteopontin immunostaining was associated with advanced nuclear grades and advanced stages of ductal adenocarcinomas (43). In fine needle aspirations, abundance of the splice variants OPN-b and OPN-c indicated a poor prognosis. While OPN-b was a predictor for survival, OPN-c was associated with metastatic disease (73).

iii) A low stromal deposition of osteopontin was associated with a poor survival, independently of established prognostic factors (47).

iv) In ductal adenocarcinoma, osteopontin staining was not associated with grade and stage (44). In stage-oriented pancreas cancer tissue arrays, high and preferentially cytoplasmic osteopontin staining in 80% of carcinomas did not correlate with tumor stage (74). Osteopontin mRNA levels in ductal adenocarcinoma, adjacent non-malignant and benign pancreatic tissues highly correlated with each other, but did not correlate with venous lymphatic invasion, diabetes, obesity, T stage, body mass index or survival (68).

v) The presence of osteopontin in adenocarcinoma may have a protective effect, independently of tumor stage (45).

The infrequent presence of psammoma bodies in pancreatic cancer may be associated with a less aggressive tumor phenotype, potentially leading to a better prognosis. In very rare cases of ductal adenocarcinomas, focal dystrophic calcification may arise. In one case, numerous psammoma bodies, scattered throughout the tumor, were positive for osteopontin. Due to its high calcium-binding capacity, osteopontin can play a role in the development of such psammoma bodies. Calcifications in imaging may be early indicators of cancer (75). In a case of intraductal tubulopapillary neoplasm with severe calcification, psammoma body-type and non-psammoma body-type calcifications stained positively for osteopontin, the macrophages were weakly positive, and the tumor cells were also stained strongly (76).

*Survival*. While (as with progression) published studies on survival are not consistent, there is a preponderance of findings that osteopontin, particularly its splice variants, prognosticates a poor outcome.

i) In bulky ampullary cancer, osteopontin-positive infiltrating tumor-associated macrophages and the expression of macrophage migration inhibitory factor (MIF) have been found to be associated with the worst disease-specific survival (63). In fine needle aspirations, the abundance of the splice variants OPN-b and OPN-c indicated a poor prognosis, with OPN-b being a predictor for survival. In comparisons between long and short postsurgical survival of adenocarcinoma, RNA for total osteopontin, as well as the splice variants OPN-b and OPN-c, were more frequently expressed in short-term survivors (58,73).

ii) Although osteopontin and RAN mRNA levels in ductal adenocarcinoma highly correlated with each other, the levels of either did not correlate with invasion or survival (68). In an exploration of seromarker levels for outcomes in locally advanced cancer, patients who received chemotherapy and stereotactic body radiation therapy showed no association of osteopontin with improved survival (77).

iii) The median and 2-year overall survival was longer when osteopontin was expressed in pancreatic cancer. Osteopontin expression and T stage were independent predictors of overall survival, while other histopathologic factors (tumor grade, tumor size, nodal status) were not (45). A low stromal deposition of osteopontin correlated with a poor survival, independently of established prognostic factors for pancreatic cancer (47).

### Tumor blood. Protein biomarker

The diagnostic and prognostic value of blood osteopontin (Table SIV) has been corroborated by various studies (78). Overall, diverse reports have been in good agreement.

i) In profiling plasma biomarkers in pancreatic cancer, osteopontin was detected at twice the level compared to healthy controls (79) and higher in patients with ductal adenocarcinoma compared with chronic pancreatitis patients, type 2 diabetes and healthy controls (80). Osteopontin was substantially upregulated in late-stage ductal adenocarcinoma (81); however, alone, it was not a marker for obesity or progenitor cell trafficking (82).

ii) Gastro-entero-pancreatic neuroendocrine tumors are highly vascularized neoplasms. While the plasma concentrations of TIE-2 and CgA were higher in patients (2 of 16 pancreatic) as compared to healthy controls, the osteopontin values were not significantly elevated (possibly due to a lack of power) (83).

iii) In a previous meta-analysis of serum levels for the diagnosis of pancreatic cancer, osteopontin was higher in patients than in healthy controls. Ethnicity-stratified analysis indicated that this elevation occurred among both Caucasians and Asians (84). Serum osteopontin levels were elevated in patients with resectable adenocarcinoma compared to healthy individuals (42). In an expanded comparison of preoperative patients with resectable adenocarcinoma, compared to patients with chronic pancreatitis and healthy controls, osteopontin distinguished pancreatic cancer vs. chronic pancreatitis or healthy controls (85). Osteopontin performed very highly in distinguishing pancreatic cancer cases from healthy people. The sensitivity dropped precipitously when tested on an expanded set of controls, including patients with pancreatitis (86). ELISA of serum from patients with adenocarcinoma, patients with chronic pancreatitis and healthy donors revealed a 1.6-fold increase in osteopontin serum levels in patients with tumors and a 1.9-fold increase in patients with chronic pancreatitis (48). Depending on the cut-off value, the sensitivity in these studies varied over some range, but the specificity was always >90%.

iv) Mean pre-operative serum osteopontin levels in patients with ampullary neoplasms (vs. patients with other periampullary diseases and healthy controls) were elevated (61).

*RNA biomarker.* By reverse transcription-PCR from whole blood, OPN-b and OPN-c were elevated in patients with pancreatic cancer as compared to healthy controls (87). This was consistent with protein measurements in sera. No OPN-b or OPN-c was detected in healthy sera. In patients with pancreatic lesions (comprising ductal adenocarcinoma and IPMN), OPN-b was expressed in almost 50%, OPN-c in 35%, and both in 5% (88).

Gene expression alterations indicative of pancreatic cancer can be detected by profiling the RNA of pancreatic juice. In cancer patients, it contained increased levels of osteopontin, as well as IL-8, interferon-induced transmembrane protein 1 (IFITM1), fibrinogen, the chemokine CXCR4, decay-accelerating factor (DAF or CD55) and nicotinamide N-methyltransferase (NNMT) (89).

*Marker combination.* Information on markers is presented in Table SIII. Osteopontin shares characteristics of adipokines and holds the promise of being complementary to the glycoprotein tumor marker CA19-9 as an early clinical diagnostic marker (38). Its combination with CA19-9 improved differentiation over either marker alone (80). In ductal adenocarcinoma, there was a potential benefit of using osteopontin, CA19-9, and the metalloproteinase inhibitor TIMP-1 in a panel (90). A 6-plex immunoassay, including osteopontin, analyzed early- and late-stage adenocarcinoma vs. intraductal papillary mucinous neoplasms (IPMN), pancreatitis and healthy controls, osteopontin outperformed CA19-9 in separating IPMN from chronic pancreatitis (91). In pre- and post-surgical samples, a postoperative increase in plasma osteopontin raised the hazard for poor survival. Carcinoembryonic antigen (CEA) levels correlated with those of osteopontin (49). Strong correlations with osteopontin existed for the cancer markers CEA, C-reactive protein (CRP) and CA72-4. Osteopontin levels also positively correlated with the liver function readouts bilirubin, AST, GGT and ALP. A negative correlation was present between osteopontin levels and albumin and HDL-cholesterol. Levels of cholesterol, LDL, triglycerides, HOMA-IR and glucose did not correlate with osteopontin concentrations (80). MIF and osteopontin performed very highly in distinguishing pancreatic cancer cases from healthy controls. The sensitivity dropped when the set of controls was expanded to include patients with pancreatitis (86). In a conflicting report, an ELISA comparison of preoperative serum from patients with resectable adenocarcinoma, as well as sera from patients with *chronic pancreatitis* and healthy controls, osteopontin did not provide additional diagnostic power to the independent predictors of diagnosis, MIC-1 and CA19-9(85).

*Progression*. Previously, using ELISA, a post-operative increase in 10 ng/ml plasma osteopontin elevated the hazard for reduced survival (49). Although patients with ductal adenocarcinoma in stage IV had higher osteopontin plasma levels than patients in stage II, there was no difference in the levels of stage III compared to stage II (80). In a 6-plex immunoassay, osteopontin aided the delineation of cancer from benign lesions and healthy controls, but it did not change significantly with stage (91).

*Post-treatment recovery*. Post-operative stress was assessed in serum from patients following hepatobiliary pancreatic cancer surgery without post-operative complications. While the low-stress group did not exhibit significant increases in the levels of osteopontin on post-operative periods, the medium- and high-stress groups did, which peaked on post-operative day 3(92). Plasma osteopontin levels were not different between pre- and post-surgical specimens, but both were significantly elevated over controls (49). Osteopontin levels in serum samples, taken from the same patients before and 6 days after pancreatic resection, displayed a decrease in ~30%, an increase in ~40%, and a smaller than 20% change in ~25% (48).

Circulating biomarkers have been correlated with efficacy and tolerability to antiangiogenic agents, such as sunitinib. Osteopontin was associated with shorter progression-free survival, independently of Ki-67. Its levels remained higher after 6 months of treatment in non-responders than in responders (93). The combination of galunisertib, the first small-molecule TGFβ Receptor inhibitor, with gemcitabine has resulted in the improvement of survival in patients with unresectable pancreatic cancer. In multi-marker analysis from patient plasma, baseline proteins that were changed during treatment included osteopontin, amphiregulin, the cancer marker CA15-3, cathepsin D, P-Selectin, RAGE, sortilin, cartilage oligomeric matrix protein (COMP), eotaxin-2, N-BNP and thrombospondin-4(94). In another study which explored seromarker levels for associations with outcomes in locally advanced cancer, patients who received chemotherapy and stereotactic body radiation therapy showed no association of osteopontin with improved survival (77).

### Tumor immunology

Osteopontin is expressed in tumor-infiltrating immune cells (Table SV). Macrophages are prominently involved.

i) Adenocarcinoma contained strong osteopontin mRNA signals in tumor-infiltrating macrophages in close to 60% of specimens, while its expression was not detectable in healthy pancreatic tissue or in the macrophages distant from the infiltrating cancer (42).

ii) In a comparison between ductal adenocarcinoma and undifferentiated carcinoma, osteopontin was expressed, apart from the tumor cells, in macrophages and osteoclast-like giant cells. There was no correlation between the number of osteopontin-positive macrophages and tumor cells (54). Occasional peritumoral inflammatory cells (macrophages) exhibited osteopontin positive stain (74). M2 macrophages were significantly accumulated. In cellular communication, the osteopontin-CD44 pathway between macrophages and epithelial cells was particularly strengthened in ductal adenocarcinoma (compared to IPMN) (72).

iii) A 5-gene immune-related signature, including osteopontin, SNHG10, CASC19, LINC00683 and LINC00237 enabled the development of a risk score formula to predict the overall survival of ductal adenocarcinoma patients, as well as a nomogram, combining risk score, N stage, and margin status. The expression level of osteopontin, mainly in ductal cells and macrophages, was related to prognosis and immune regulators (95).

iv) Tumor-associated macrophages promote cancer cell proliferation and distant metastases. They often overexpress osteopontin. Osteopontin levels and location of tumor-associated macrophages in bulky ampullary cancer predicted recurrence. Patients with bulky tumor, osteopontin-positive infiltrating tumor-associated macrophages and MIF-expression had the worst disease-specific survival (63).

The abundance of osteopontin in tumor-associated macrophages may serve as a poor prognostic indicator.

Fibroblasts and macrophages are heterogeneous cell populations able to enhance metastasis by ductal adenocarcinoma. Mesenchymal stem cells can be recruited by osteopontin from either peripheral blood or bone marrow. Tumor-localized fibroblasts may be reprogrammed by osteopontin to become cancer-associated fibroblasts, as may be M1 anti-tumor macrophages, which are reprogrammed to become tumor-associated macrophages (38). Metastasis-associated fibroblast heterogeneity in the liver is regulated by macrophages via 3 functionally distinct subpopulations, among which the generation of pro-metastatic and immunoregulatory myofibroblastic metastasis-associated fibroblasts critically depends on the macrophages. This subset was induced through a STAT3-dependent mechanism, driven by macrophage-derived progranulin and cancer cell-secreted leukemia inhibitory factor (LIF). In a reciprocal manner, osteopontin secreted from myofibroblastic metastasis-associated fibroblasts promoted an immunosuppressive macrophage phenotype, resulting in the inhibition of cytotoxic T-cell functions (Fig. S3). The blockade or depletion of STAT3 restored an antitumor immune response and reduced metastases (96).

PD-L1 is expressed in pancreatic cancer cells, myeloid-derived suppressor cells, polymorphonuclear myeloid-derived suppressor cells and tumor-associated macrophages. Despite cytotoxic T-lymphocyte infiltration in the tumor microenvironment, pancreatic cancer stands out as one of the malignancies responding poorly to immune checkpoint blocker therapy. In non-response to treatment, epigenomic dysregulation has emerged as a mechanism of T-cell exhaustion. Mouse pancreatic tumors have a genome-wide increase in H3K4me3 deposition (an epigenetic modification to the DNA packaging protein Histone H3 that indicates tri-methylation at the 4th lysine residue of the histone H3 protein) as compared with healthy pancreas. Upstream, WD-repeat domain 5 (WDR5) is essential for H3K4me3-specific histone methyltransferase activity. Downstream, osteopontin and its receptor CD44v are upregulated by their promoter H3K4me3 deposition and are primarily expressed in tumor cells and monocytic myeloid-derived suppressor cells. Osteopontin may compensate PD-L1 function to promote pancreatic cancer immune escape. Pharmacological inhibition of the epigenetic WDR5-H3K4me3 axis is effective in suppressing pancreatic tumor immune escape and in improving efficacy of anti-PD-1 immunotherapy in pancreatic cancer (97).

Leukocytes can be recruited by osteopontin from either peripheral blood or bone marrow (38). Notably, a proinflammatory immune component was distinctly present in intraductal papillary mucinous neoplasms, comprising CD4^+^ T-cells, CD8^+^ T-cells and B-cells. Osteopontin is a biomarker for the surveillance of carcinogenic progression (72).

### Tumor models

**Figure 2:**
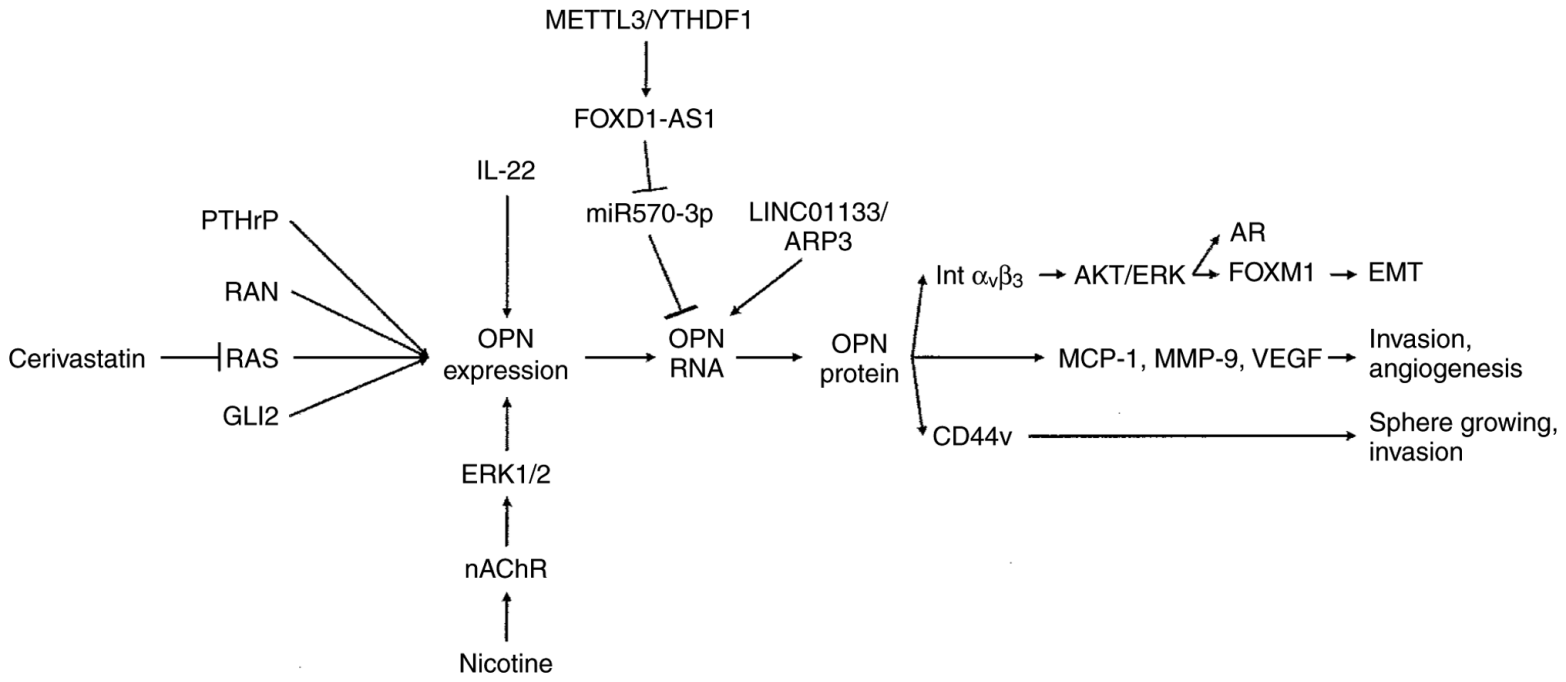
Osteopontin signaling in pancreatic cancer. In pancreas cancer, several signals can induce Osteopontin expression (left portion), including environmental exposure (bottom portion). mRNA stabilization may increase Osteopontin (top portion). The elevated levels lead to tumor progression, predominantly via autocrine or paracrine effects through the receptors integrin α_V_β_3_ and CD44v (right portion). Osteopontin splicing is not depicted, because the underlying molecular processes have not been elucidated.

Osteopontin is integrated into a network of signal transduction, which results in the activation of functional changes that support cancer progression, including epithelial-mesenchymal transition (mainly through integrin α_V_β_3_), sphere growing and invasion (mainly through CD44v), and the secretion of soluble mediators (MCP-1, MMP-9 and VEGF) (Fig. 2).

*Metastasis*. Osteopontin promotes epithelial-mesenchymal transition and cancer stem cell-like properties.

i) Constant suppression of BMP activity by its antagonist gremlin 1 (GREM1) is essential for maintaining the fate of epithelial ductal adenocarcinoma cells.

ii) Osteopontin is an essential regulator of mesenchymal cell fate.

Osteopontin, secreted from epithelial cells bound to integrin β_3_ on mesenchymal ductal adenocarcinoma cells to induce BMP2 and GREM1 expression. GREM1 inhibition of BMP signaling was required for osteopontin expression in epithelial cells, thereby forming an intercellular regulatory loop. Mesenchymal and epithelial ductal adenocarcinoma cell fates are determined by the reciprocal paracrine regulation of the soluble factors GREM1 and osteopontin (81). Long non-coding RNAs (lncRNAs) are involved in the tumorigenesis and progression of ductal adenocarcinoma. Osteopontin is regulated by some of them. LINC01133 is one of 16 hub genes that can predict prognosis. Its overexpression compared to adjacent tissue may promote proliferation and metastasis as well as inhibit apoptosis. Expression of LINC01133 and osteopontin were positively correlated, leading to enhanced epithelial-mesenchymal transition. LINC01133 bound to actin-related protein 3 (ARP3), and this complex reduced osteopontin mRNA degradation, allowing for increased osteopontin mRNA levels (98). Pancreatic stellate cells (myofibroblast-like cells that are located in exocrine regions of the pancreas) in the tumor microenvironment contribute to invasion and metastasis. Osteopontin was highly expressed and secreted, driven by hypoxia in a reactive oxygen species-dependent manner. Signaling through integrin α_V_β_3_ was involved in the epithelial-mesenchymal transition (65). Upon stimulation with certain cytokines and accompanied by the increased production of osteopontin, pancreatic stellate cells can be activated into cancer-associated fibroblast (CAF) isoforms, such as inflammatory CAF and myofibroblastic CAF, which participate in the desmoplastic reaction to remodel the mesenchyme of pancreatic cancer (38).

Osteopontin increased the invasiveness of pancreatic cancer cells, without having any impact on cell proliferation. The effect was dependent on the receptor CD44(48). Cancer stem-like cells have increased capacity to invade and grow as spheres in a manner that requires CD44v6. They displayed elevated expression of markers for metastases, including osteopontin, CD44v6, and the chemokine CXCR4, when compared with their adherent counterparts (99). In cancer stem cells, FOXD1-AS1 was upregulated. There, it promoted tumorigenesis and self-renewal. It did so by upregulating osteopontin and acting as a ceRNA to sponge miR-570-3p (88). The overexpression of the splice variants OPN-b and OPN-c in ductal adenocarcinoma cells increased their activity in soft-agar colony formation and wound healing assays, induced the transcription of interleukin-6, and reduced tumor necrosis factor-α (TNF-α), interferon-γ and IL-10(88). One conflicting study was unable to detect any osteopontin protein in the in the supernatants or by western blotting in the lysates of three commonly used ductal adenocarcinoma cell lines studied (44).

Osteopontin has long been known to be a downstream target of RAS signaling (9,100). PTHrP is frequently amplified as part of the KRAS amplicon in patients with pancreatic cancer. It is highly enriched in ductal adenocarcinoma metastases, and its upregulation drives the growth of both primary and metastatic tumors. The osteopontin gene was a downstream effector of PTHrP, overexpression, which enhanced migratory capacity and metastatic ability (101). The small GTPase RAN drives pancreatic cancer metastasis by modulating AR expression. While RAN plays physiological roles in the regulation of nuclear transport and microtubule spindle assembly, it also mediates the invasive and liver-metastatic functions of osteopontin. In RAN-silenced cells, osteopontin was necessary and sufficient to restore AR levels via the PI3K/AKT signaling pathway. AR reversed the inhibitory effects of RAN silencing or osteopontin silencing on the mobility and invasion of cancer cells. However, osteopontin did not have any significant effect on RAN transcription (68,69). The anti-proliferative effects of statins and hemin on pancreatic cancer cell lines do not appear to be related to the heme oxygenase pathway. While the iron-containing porphyrin hemin triggered reactive oxygen species-induced cell death, the HMG-CoA reductase inhibitor cerivastatin targeted RAS protein trafficking and affected markers of invasiveness. Osteopontin mRNA expression was significantly suppressed at 12 h of treatment with persisting effect of up to 48 h (102).

The zinc-finger DNA-binding transcription factors of the GLI family are effectors of hedgehog signaling involved in cell fate determination, proliferation and patterning in most organs during embryo development. Sonic hedgehog (SHH)-GLI1 signaling and osteopontin play vital roles in ductal adenocarcinoma. Proliferation, migration and invasion were decreased, whereas apoptosis was increased, when GLI1 or osteopontin was knocked down. Exogenous osteopontin protein could partially reverse the effect of both osteopontin and GLI1 knockdown. GLI1, but not SHH, was associated with osteopontin expression, and GLI1 regulated osteopontin production through a non-canonical pathway that did not utilize smoothened (SMO). Hedgehog signaling promotes proliferation, migration and invasion, but inhibits apoptosis of pancreatic cancer cells through the upregulation of osteopontin (55). Ductal adenocarcinoma is a heterogeneous disease, comprised of a classical and a basal-like subtype. These subtypes are not permanently encoded, but the transcription factor GLI2 is a master regulator of their inter-conversion. Its activation was sufficient to convert classical to basal-like phenotypes. GLI2 upregulated the expression of osteopontin, which was critical for metastatic growth and adaptation to oncogenic KRAS ablation. Accordingly, elevated GLI2 and osteopontin levels predicted shortened overall survival. Thus, the GLI2-osteopontin circuit is a driver of ductal adenocarcinoma cell plasticity that establishes and maintains an aggressive variant of this disease (103).

*Tropism.* From the parental pancreatic cancer cell line HPC-4, two sublines were selected, resulting in a liver metastatic cell line and a peritoneal disseminated cell line. The liver metastatic cells expressed elevated levels of IL-8 and integrin α_V_β_5_. Among upregulated genes in liver-tropic cells compared with peritoneum-tropic cells were οsteopontin, VEGF and hepatocyte growth factor (HGF) (104). Osteopontin was upregulated in the liver-metastatic cell line in comparison to the parental cells. Inhibition by micro-RNA or antibody significantly reduced the metastatic rate (105).

Osteopontin mRNA in a panel of human pancreatic cancer cell lines was significantly related to their growth in the liver of nude rats. In co-culture of cancer cells with hepatocytes, οsteopontin mRNA was increased in the tumor cells, and its downregulation was associated with reduced cell proliferation (106,107). From a panel of pancreatic cancer cell lines, some had the properties to grow in the liver of rats and mimic liver metastasis of ductal adenocarcinoma. Among 33 associated genes and 5 signaling pathways, οsteopontin, MMP-1 and IGF-1 stood out (108).

In the liver, macrophages regulate metastasis-associated fibroblast heterogeneity via distinct subpopulations. The generation of pro-metastatic and immunoregulatory myofibroblastic metastasis-associated fibroblasts critically depended on macrophages. Reciprocally, οsteopontin secreted from myofibroblastic metastasis-associated fibroblasts promoted an immunosuppressive macrophage phenotype (96).

*Stemness.* Cancer stem cells play a pivotal role in the pathogenesis of human malignancies. Pancreatic cancer stem-like cells have the capacity to grow as spheres and display increased invasion capability. The sphere-growing population is not only composed of cells expressing classical stem membrane markers, but also needs CD44v6^+^ cells. The stem-like cells are distinguished by upregulated expression of markers for metastasis, including οsteopontin and CXCR4, when compared with their adherent counterparts (99).

Cancer stem cells are considered responsible for the recurrence of cancer. Dysregulated autophagy is highly prevalent in many malignancies and has been implicated in cytoprotection and tumor promotion. Induction of autophagy, mediated by οsteopontin/NF-κB signaling, is required for the maintenance of pancreatic cancer stem cell activity (109).

lncRNAs play a role in modulating cancer stemness features. Specifically, in this subpopulation, the lncRNA FOXD1-AS1 is upregulated. It promotes tumorigenesis and self-renewal by acting as a competitive endogenous RNA to sponge (bind to) miR-570-3p, a microRNA that would otherwise suppress the expression of osteopontin. Thus, the production of osteopontin is upregulated. The elevated levels of FOXD1-AS1 in cancer are facilitated through METTL3 and YTHDF1-dependent m6A methylation (110).

The interplay between tumor-microenvironment factors and cancer stem cells plays critical roles in the aggressiveness of pancreatic cancer.

i) CAFs promote cancer stem cell features. The long-term treatment of pancreatic cancer cells with CAF-conditioned medium enriched stemness, as reflected in increased tumor-sphere formation and elevated self-renewal, as well as drug-resistance markers. CAFs in 3-dimensional co-culture with pancreatic cancer cells induced a substantial increase in stemness features. The expression of CD44 and α-SMA progressively increased from the early to late stages. Osteopontin was the top differentially overexpressed gene, and its knockdown reduced the stemness characteristics. There is an interplay between cancer-associated fibroblasts and enrichment of stemness population through the osteopontin/CD44 axis (111).

ii) Osteopontin from activated pancreatic stellate cells interacts with integrin α_V_β_3_ on pancreatic cancer cells to upregulate FOXM1 expression. It promotes epithelial-mesenchymal transition and cancer stem cell-like properties of the cancer cells by activating the integrin α_V_β_3_-AKT/ERK-FOXM1 cascade in a paracrine manner (65).

*Angiogenesis*. Obese visceral adipocytes trigger aggressive transformation in ductal adenocarcinoma cells to induce progression and accelerate angiogenesis via osteopontin secretion. Conditioned media from these cells increased ductal adenocarcinoma angiogenic capacity. The adipocytes directly increased the migratory, cell growth and tube-forming capabilities of endothelial cells in an osteopontin-dependent manner via increased AKT phosphorylation and VEGF-A expression in both ductal adenocarcinoma and endothelial cells. Tumor volume was increased in obese mice compared with lean mice, whereas blocking osteopontin inhibited obesity-accelerated tumor growth (112).

The tumor position, head and uncinate process, or body and tail of the pancreas, are critical for surgical strategies. Osteopontin is secreted by vessel endothelial cells in body and tail pancreatic ductal adenocarcinoma and is associated with increased tumor burden. Conversely, the number of tumor cells marked by the retinoic acid carrier protein CRABP2 was lower in body and tail pancreatic ductal adenocarcinoma and was a prognostic marker of overall survival (113).

*Chemoresistance.* Osteopontin-induced autophagy via activation of the NF-κB pathway contributes to chemoresistance against gemcitabine in cancer cell lines. By silencing osteopontin expression, gemcitabine conferred enhanced cytotoxic effects. This implies that the combination of gemcitabine with pharmacological autophagy inhibitors is a promising therapeutic strategy (109).

A low level of FOXD1-AS1 may serve as a predictor of 5-fluorouracil benefits in cancer patients. Pancreatic cancer cells depleted of lncRNA FOXD1-AS1 exhibit heightened sensitivity to 5-fluorouracil-indued cell growth inhibition and apoptosis. The introduction of osteopontin could reverse the sensitivity of long non-coding RNA FOXD1-AS1-knockdown cancer cells to 5-fluorouracil-induced cell apoptosis (110).

Bis-indole derivatives and substituted quinolines exhibit anti-inflammatory activities. While both classes of drugs are nuclear receptor 4A2 (NR4A2, NURR1) ligands, they interact differentially with their drug target. The activation of gene expression, including osteopontin, by bis-indole and quinoline-derived activators of nuclear receptor 4A2 was structure-dependent. These compounds induced osteopontin expression at variable potency in distinct pancreatic cancer cell lines (114).

Pancreatic stellate cells in the tumor microenvironment contribute to chemoresistance. Osteopontin is highly expressed and secreted by activated pancreatic stellate cells (65).

### Predisposition and early lesions. Prediagnosis and premalignancy

Biomarkers were evaluated in prediagnostic sera (collected before these patients had been clinically diagnosed with pancreatic cancer) obtained from cases of pancreatic cancer enrolled in a large prospective study. The panel of osteopontin, CA19-9 and OPG, identified in a prior retrospective study, was not effective (115). However, a 6-plex immunoassay (including osteopontin) analyzed adenocarcinoma vs. intraductal papillary mucinous neoplasm, pancreatitis and healthy controls. It distinguished adenocarcinoma from non-cancer conditions and outperformed CA19-9 in separating intraductal papillary mucinous neoplasms from chronic pancreatitis (91). Intraductal papillary mucinous neoplasms of the pancreas are bona fide precursor lesions for ductal adenocarcinoma. According to a comparison between high-grade IPMNs and IPMN-derived ductal adenocarcinomas, there were heterogeneous alterations within the epithelium and the tumor microenvironment during the progression of non-invasive dysplasia to invasive cancer. For the epithelial cell component, there existed acinar-ductal cells and isthmus-pit cells enriched in precursor lesions. Osteopontin was a biomarker for the surveillance of carcinogenesis by intraductal papillary mucinous neoplasms (72).

*Smoking.* Smoking substantially increases the risk of developing pancreatic cancer and accounts for up to 25% of cases. In cancer tissue from patients with invasive ductal adenocarcinoma, the majority of whom were smokers, there were significant amounts of osteopontin in the malignant ducts and the surrounding pancreatic acini (56). The splice variant OPN-c was present in almost 90% of lesions, of whom three fourth were smokers. The levels of OPN-c correlated well with higher expression levels of total osteopontin in tissue and serum from patients (59,60). The nicotine receptor was expressed in the invasive and premalignant lesions without clear correlation with smoking history (60).

Cigarette smoke and nicotine are among the leading environmental risk factors for developing ductal adenocarcinoma. In a previous study, exposure to cigarette smoke caused an increase in pancreatic osteopontin that paralleled the rise of pancreatic and plasma nicotine levels (56). Nicotine activated the osteopontin promoter through the α7-nicotine acetylcholine receptor (α7-nAChR) via the activation of ERK1/2 (but not p38 or c-Jun NH2-terminal MAP kinases) in ductal adenocarcinoma cells (56,60). While these cells expressed varying levels of OPN-a, OPN-b, and α7-nAChR (67), their nicotine treatment selectively induced the *de novo* expression of the splice variant OPN-c (59) and increased α7-nAChR levels (60).

Smoking and nicotine may also contribute to ductal adenocarcinoma metastasis by inducing MMP-9 and VEGF expression. Osteopontin plays a central role in mediating these effects via promoter activation (66). OPN-c induced MCP-1 promoter activity and increased its mRNA and protein. Through this chemokine, nicotine may contribute to ductal adenocarcinoma inflammation. MCP-1 was present in 60% of invasive lesions, of whom two thirds were smokers (67).

*Pancreatitis.* During repeated or long-term inflammation, cytokines and reactive oxygen species can cause DNA damage, predisposing to cancer (40). Persistent pancreatitis is linked with a substantially increased risk for pancreas cancer. Osteopontin was elevated in pancreatitis, increasing progressively from normal to recurrent acute pancreatitis and chronic pancreatitis (116).

Plasma osteopontin levels could serve as a highly sensitive and specific early marker of mortality in patients with acute pancreatitis. Its abundance was elevated, and at admission it could uniquely predict mortality (117). Serum osteopontin levels increased and osteocalcin levels decreased in the course of acute pancreatitis in critically ill patients with systemic inflammation. Osteopontin prognosticated the development of organ failure, even after adjustment for high-sensitivity C-reactive protein and demographic parameters, with its accuracy being similar to that of the more complex APACHE II score (118). None of the bone biomarkers were significantly parted between patients with alcohol-induced acute pancreatitis and other etiologies, thus likely not reflecting a potentially inferior nutritional status and impaired bone turnover in subjects with alcohol-induced acute pancreatitis. Early in the course of acute pancreatitis, osteopontin may help decide, who will benefit from closer monitoring and aggressive therapy (118).

In fine needle aspirates, OPN-a was expressed in 50% of patients with chronic pancreatitis, OPN-b in <20%, whereas OPN-c was totally absent (73). There was a 1.6-fold increase of osteopontin mRNA in chronic pancreatitis samples compared to healthy pancreatic tissues (48). In a separate study, osteopontin levels were increased in patients with chronic pancreatitis in comparison with those with type 2 diabetes and healthy controls. Osteopontin was lower in chronic pancreatitis patients, type 2 diabetes and healthy controls compared with patients with ductal adenocarcinoma. At a defined cut-off, osteopontin differentiated chronic pancreatitis from ductal adenocarcinoma with acceptable sensitivity and high specificity. The combination of osteopontin with CA19-9 brought about a better differentiation. There were no significant differences in osteopontin concentrations among chronic pancreatitis patients categorized according to the CP stage (80). A 6-plex immunoassay, including osteopontin, analyzed pancreatitis vs. adenocarcinoma, IPMN and healthy controls. Osteopontin outperformed CA19-9 in separating chronic pancreatitis from IPMN (91).

Cytokines are key mediators of inflammation. They contribute to pancreatitis pathophysiology via several mechanisms:

i) IL-22 acts primarily on epithelial and stromal cells to protect against apoptosis, stimulate proliferation of epithelial cells to repair injured tissues, and induce the expression of antimicrobial peptides (including the REG family, see type 2 diabetes below). Acinar cells respond to IL-22 with activation of STAT3 and changes in gene transcription. IL-22 signals through a receptor comprising IL-10Rβ (CRF2-4) and IL-22R. Whereas IL-10Rβ exhibits a broad distribution of expression, IL-22R follows a restricted expression pattern, with the highest levels in pancreas and detectable expression in multiple other tissues. IL-22 mediated robust inductions of expression for osteopontin and pancreatitis-associated protein 1 (PAP1) through IL-10Rβ. It may play a role in the pancreatic immune response (119).

ii) The transplantation of islets of Langerhans is a potentially curative treatment for diabetes. Ensuing immunosuppressive regimens have had some success in achieving insulin independence. Nevertheless, transplanted islets are exquisitely susceptible to the injurious effect of mediators elicited by a very early host inflammatory response, which results in islet cell dysfunction and possibly death of the transplanted tissue. Pro-inflammatory cytokines and macrophage-derived IL-1β, TNF-α, and nitric oxide perturb insulin secretion from β-cells and whole islets. Osteopontin administration dose-dependently improved islet cell-derived glucose-stimulated insulin secretion and inhibited IL-1β-induced nitric oxide production in an arginine-glycine-aspartate-dependent manner (the RGD motif is important for integrin binding). The protective effect was accompanied by inhibited transcription of iNOS and reduced activation of NF-κB, resulting in decreased formation of the toxic nitric oxide. Islets exposed to IL-1β revealed a naturally occurring early upregulated osteopontin transcription, suggesting the presence of a cross-talk between the IL-1β and osteopontin pathways (120).

iii) While serum osteopontin was only marginally increased in pancreatitis, gastrointestinal or muscle damage, it was increased in liver and (to a lesser extent) kidney damage. Biomarkers of tissue injury include osteopontin, GLDH, K18 and ccK18 (intact and caspase-cleaved cytokeratin-18), MCSF, MCSFR, ALT and miR-122(121).

Obesity increases the severity of acute pancreatitis. The PPAR-γ agonist rosiglitazone serves as a treatment choice for the disease.

i) Rosiglitazone, in mice without acute pancreatitis fed both a low-fat diet and high-fat diet, increased body weight and percent fat mass, with the upregulation of adiponectin and the suppression of erythropoiesis.

ii) In mice with acute pancreatitis fed a high-fat diet, rosiglitazone increased survival and hastened recovery from pancreatic inflammation. This was associated with lower circulating and pancreas-associated levels of osteopontin, IL-6, galectin-3 and TIMP-1, particularly within one week post-acute pancreatitis.

iii) In mice with acute pancreatitis fed a low-fat diet, rosiglitazone worsened the degree of intrapancreatic acinar and fat necrosis as well as visceral fat saponification, without affecting other parameters of disease severity or inflammation (122).

Due to its high calcium-binding capability, osteopontin maintains the homeostasis of this electrolyte. The deregulation of osteopontin levels in disease may be associated with calcifications.

i) Autoimmune pancreatitis has the potential for calcification over a long-term clinical course. In surgical specimens from autoimmune pancreatitis, chronic pancreatitis, and healthy pancreas, as well as autoimmune pancreatic tissues from rats, the incidence of osteopontin expression in centroacinar cells in chronic pancreatitis with calcification and in autoimmune pancreatitis was greater than that in healthy controls. Some cases of autoimmune pancreatitis and chronic pancreatitis expressed the receptor CD44 in centroacinar cells and ductal cells. The inflammatory area and percentage of osteopontin/CD44-positive cells increased with advancing age (123).

ii) Osteopontin is absent from the acinar or ductal cells of the healthy pancreas. However, its RNA is detectable in all cases of chronic calcifying pancreatitis and in over half of cases of chronic pancreatitis without stones. The molecule may play an important role in stone formation, and its targeting could lead to an effective therapeutic approach to the inhibition of calcification associated with chronic pancreatitis (124).

*Kidney disease*. An increased osteopontin mRNA and protein expression correlated with proteinuria, reduced creatinine clearance, and kidney fibrosis. In patients with chronic kidney disease, there was a positive colocalization of the osteopontin association signal at SPP1 with pancreas tissue. Downstream analyses revealed colocalization of the osteopontin association signal at KLKB1 (prekallikrein) with various plasma proteins in trans, and with phenotypes (bone disorder, deep venous thrombosis) (125).

The microangiopathy of diabetes may lead to renal damage, for which the cysteine protease inhibitor cystatin C is a biomarker. Urinary levels of cystatin C were increased in diabetic fatty rats before the histopathological appearance of kidney injury, and then further increased with the progression of disease. In addition, urinary osteopontin, β_2_-microglobulin, clusterin, the glutathione transferase GSTµ and kidney injury molecule-1 (KIM-1) had the potency to detect renal damage (126).

Post-transplant diabetes mellitus is a complication occurring following kidney transplantation, caused by increased insulin resistance from glucocorticoids and decreased insulin secretion from calcineurin inhibitors. Adverse outcomes include reduced graft survival, heightened cardiovascular mortality and an elevated risk of postoperative infections.

i) The long-term administration of the immunosuppressant cyclosporine A causes hypoxic injury to the kidneys with apoptotic cell death in renal tubular cells. It is associated with renal tubular atrophy and the loss of tubular mass, leading to progressive renal failure and irreversible renal striped interstitial fibrosis. Mediators, including osteopontin in conjunction with angiotensin II and TGF-β1, are involved in the pathogenesis of chronic cyclosporine A nephropathy. Rosiglitazone had a protective effect against pancreatic and renal injury caused by cyclosporine A. It decreased blood glucose concentration, increased plasma insulin concentration and preserved pancreatic β-islet mass. The pro-inflammatory and pro-fibrotic molecules also decreased (127).

ii) Cyclosporine a exerts a diabetogenic effect by damaging pancreatic islet cell integrity. Continuous erythropoietin receptor activator (CERA) mediates tissue-protective and anti-inflammatory effects in various settings of organ injury. Its low-dose administration did not lead to improved kidney function, but it showed a trend toward upregulation of osteopontin, accompanied by increased renal macrophage-infiltration, and enhanced parenchymal TGF-β1 and IL-10. Moreover, CERA-treated animals showed amelioration of pancreatic islet cell injury (128).

In an evaluation of markers for renal graft dysfunction in patients with type 1 diabetes mellitus following kidney transplantation and simultaneous pancreas-kidney transplantation, osteopontin was significantly elevated compared to a control group of type 1 diabetes without diabetic nephropathy (129).

### Diabetes and obesity

Osteopontin is an islet protein and a pro-survival factor that is protective against both cytotoxicity and hyperglycemia, and may serve as an intrinsic feedback regulator of nitric oxide signaling in β-cells. Its expression increases with age. This phenomenon in aging islets could also affect the disease course in diabetes (51,130). Pancreatic duct epithelial cells, exposed to several combinations of glucose and insulin, displayed accelerated proliferation under high glucose culture concentrations and was more prominent in the presence of high insulin. The expression of osteopontin mRNA was also increased. Furthermore, the cells show increased oxidative stress (according to the expression of 8-OHdG DNA) in the presence of high glucose (131).

*Prediabetes.* High levels of pancreatic osteopontin mRNA and protein arose in prediabetic non-obese pancreata, entailing co-localization of osteopontin with most of the islet hormones. Transcripts for osteopontin were upregulated in the pancreatic lymph nodes at the pre-diabetic stages, commencing after the exposure of native normoglycemic non-obese predisposed islets and β-cells to a high-dose combination of IL-1β, TNF-α and IFN-γ. While cytokines induced the upregulation of osteopontin promoter activity within one hour, glucose induced a dose-dependent upregulation of promoter activity after 24 h. Long-term exposure to cytokines or glucose reduced osteopontin promoter activity and expression. There is a positive intrinsic mechanism that regulates pancreatic osteopontin expression. Its temporal pattern is inversely correlated with progression of insulitis and β-cell destruction. Exhaustion of the local protective osteopontin system in the islets is implicated in the associated loss of endogenous islet protection and progression of the destructive insulitis with resultant non-obese diabetes severity (130,132).

Hormones involved in bone remodeling and glucose metabolism may be skewed in prediabetes. Specifically, the bone is emerging as a key organ in the regulation of glucose metabolism. Vitamin D has been implicated in the pathogenesis of sub-inflammation, insulin-resistance and obesity. Its active form, 1,25-dihydroxyvitamin D, is the result of two hydroxylations that take place in pancreas, liver, kidney, and immune cells. It is conceivable that Vitamin D action and status in the regulation of calcium-phosphorous balance and bone metabolism may also mirror the interplay between other bone remodeling hormones such as osteopontin, osteoprotegerin, CX3CL1, sclerosin, and insulin in insulin-resistant states (133). In prediabetes, hormones that are involved in bone remodeling may affect glucose metabolism before overt type 2 diabetes mellitus occurs with tissue-specific mechanisms.

i) Osteopontin expression was higher in impaired glucose regulation compared to normal glucose tolerance and in isolated impaired glucose tolerance compared to impaired fasting glucose and impaired fasting glucose tolerance. Osteopontin was positively correlated with HbA1c, PTH and fasting and 2h-plasma glucose.

ii) Osteocalcin expression was similar in impaired fasting glucose and normal glucose tolerance, but lower in impaired glucose tolerance and impaired fasting glucose tolerance compared to normal glucose tolerance.

iii) Osteoprotegerin correlated with TGD/SSPI, endogenous glucose production and hepatic Insulin resistance index in impaired glucose regulation.

There was no correlation between PTH and insulin sensitivity or β-cell function parameters (134).

*Type 1 diabetes.* Random peptide libraries screening with sera from patients afflicted by insulin-dependent diabetes mellitus found five disease-specific mimotopes displayed on phages. The screen identified οsteopontin as an autoantigen of the somatostatin cells in islets. An antibody raised against the peptide corroborated the results in immunohistochemistry, western blotting and cDNA libraries screening (135). The οsteopontin protein structure in non-obese diabetes corresponds to the a-type allele of the οsteopontin gene. According to comparative analysis of the single nucleotide polymorphisms between the a-type and b-type alleles, the majority of variations are within the non-coding regions of the gene. The implication of οsteopontin in type 1 diabetes may render this molecule an early marker of the disease (132).

**Figure 3:**
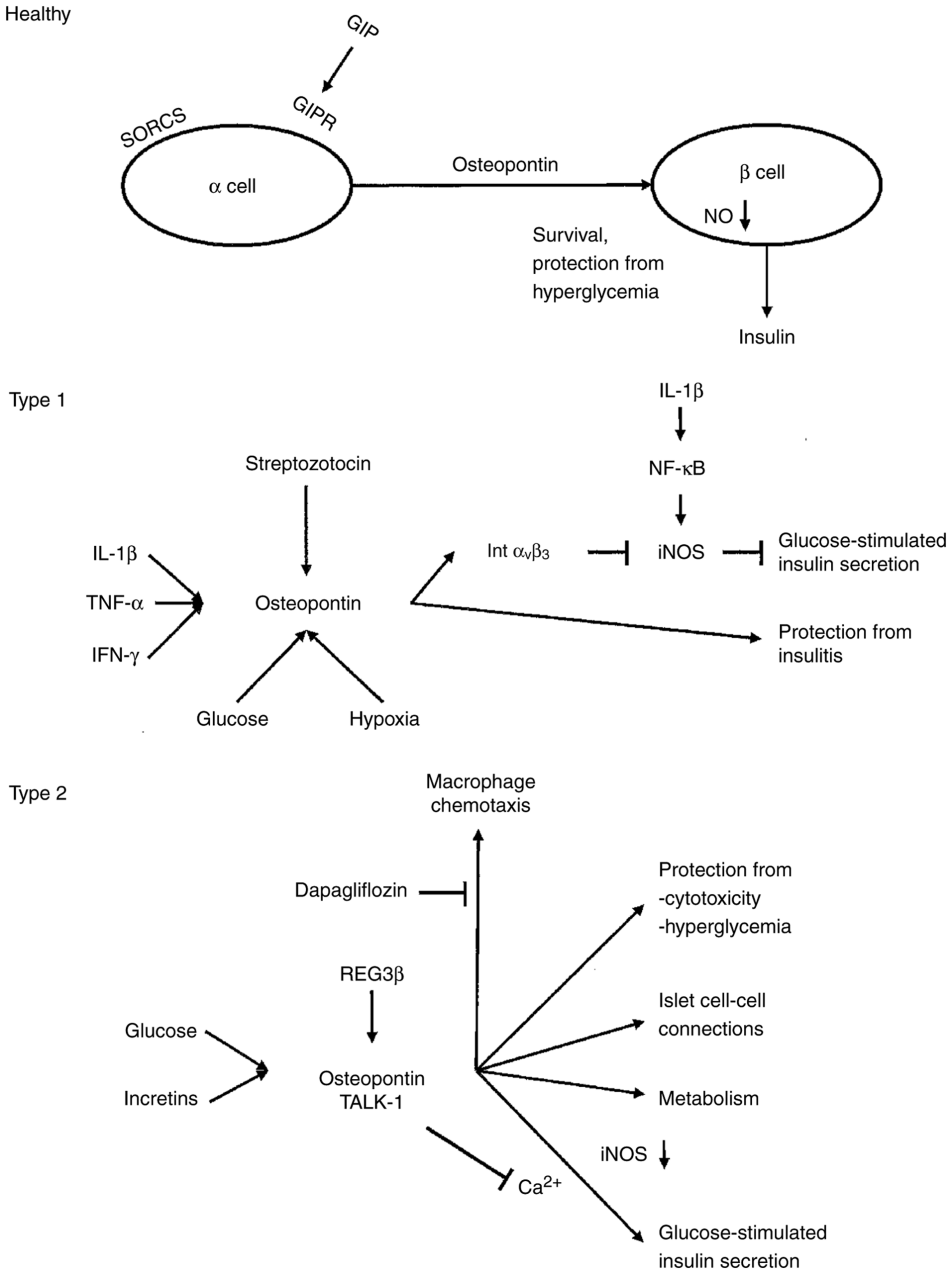
Osteopontin in diabetes. The top panel displays the physiologic role of osteopontin in the communication between pancreatic α- and β-cells. The middle panel depicts the involvement of osteopontin in type 1 diabetes. The bottom panel shows its embedding in type 2 diabetes signaling. The outcomes are listed on the right of each graph. Drug effects that have been reported are also included in the chart.

Osteopontin causes the chemotaxis of macrophages and the downregulation of nitric oxide synthesis. It acts as a regulator of the early islet autoimmune damage (Fig. 3), possibly by a shift in the steady state of type 1 diabetes pathogenesis (132,135). In the absence of osteopontin, type 1 diabetes was accelerated, suggesting a protective role of this cytokine on the insulin-producing cells of the pancreatic islets. However, there were no significant differences in osteopontin levels between patients with a duration of diabetes >3 years in comparison with those with duration <3 years (80).

The onset of type 1 diabetes is preceded by a pre-inflammatory stage, leading to insulitis. This manifestation results from the selective targeted destruction of the insulin-producing β-cells by an autoimmune phenomenon in genetically predisposed individuals. Complete β-cell destruction follows a massive invasion of the islets with a mixed population of lymphocytes and macrophages (132,136). The gene expression profiles of islets and pancreatic lymph nodes displayed consistent changes in the islets before the appearance of CD3^+^ T-cells in the islet periphery, associated with dendritic cell attraction, lymphocyte homing, and apoptosis. Whereas the level of the immunoregulatory cytokine IL-11 was low, osteopontin, IL-1 and TNFSF13B, as well as genes involved in angiogenesis were activated in the autoimmune diabetic islets (136).

The islet protein osteopontin is differentially regulated by streptozotocin and glucose (130,132). In response to streptozotocin, both wild-type and osteopontin knockout mice developed diabetes. In wild-type mice, osteopontin serum levels were upregulated within one day, mild lymphocytic infiltrate and apoptosis appeared in the diabetic islets, and upregulation of both Th1 and Th2 cytokines occurred. Pancreatic islets appeared larger in the osteopontin knockout group, no signs of inflammation arose, and Th1 cytokines were mildly upregulated with significant downregulation of IL-4. While the pancreatic immune response to multiple low-dose streptozotocin diabetes was balanced between Th1 and Th2 in wild-type animals, osteopontin knockout mice displayed mild polarization towards Th1 responses (137). Rats treated with a single dose of streptozotocin showed acute and temporary upregulation of serum osteopontin levels and pancreatic osteopontin mRNA and protein, which within a day was localized towards the periphery of the islets and surrounded the remaining insulin-positive cells. Streptozotocin induced an upregulation of osteopontin promoter activity within hours, while high glucose induced upregulation of osteopontin promoter activity after two days. Initial osteopontin upregulation after diabetes induction is likely due to streptozotocin-induced toxicity, whereas maintenance of the high osteopontin levels may be due to hyperglycemia (46).

Xenogeneic donors could potentially provide an unlimited source of islets for the treatment of type 1 diabetes. With donor xenoislet microencapsulation and host immunosuppression, adult porcine islets were able to correct hyperglycemia after intra-peritoneal transplantation in the short term. The islet xenografts lost efficacy gradually, but at graft failure, some viable islets remained, substantial porcine C-peptide was present in the peritoneal graft site, and there was very little evidence of a host immune response. Central necrosis arose in many of the encapsulated islets after graft failure, and explanted islets expressed endogenous markers of hypoxia (osteopontin, HIF-1α, GLUT-1), suggesting a role for non-immunologic factors, likely hypoxia, in graft failure. Chronic effects of non-immunologic factors, such as hypoxia and hyperglycemia, damaged the encapsulated islet xenografts (138).

*Type 2 diabetes.* Osteopontin secretion can, in a variety of situations, help cells survive an otherwise lethal insult (46). High glucose and incretins simultaneously stimulated islet osteopontin secretion, and osteopontin promoted cell metabolic activity when challenged by high glucose (139). Patients with type 2 diabetes suffer from insulin resistance and reduced insulin secretion. There is a protective role of osteopontin in pancreatic islets under diabetic conditions (Table SVI), which may point to therapeutic targets for islet protection in type 2 diabetes (139).

The osteopontin protein level, secretion and gene expression were elevated in diabetic islets. External osteopontin increased glucose-stimulated insulin secretion from diabetic, but not from healthy islets in a Ca^2+^ dependent manner (125,139). Ultra-structurally, islets reflected weaker cell-cell connections between the islet cells in the osteopontin knockout mice compared to wild-type mice. Although osteopontin knockout and wild-type β-cells have the same number of insulin granules, the former have significantly fewer docked granules. The deletion of osteopontin results in several minor β-cell defects that can be compensated for in a healthy system (140).

Epigenetics is involved in the altered expression of gene networks that underlie insulin resistance and insufficiency. Major genes controlling β-cell differentiation and function, such as PAX4, PDX1 and GLP1 receptor, are epigenetically controlled. Epigenetics can cause insulin resistance through immunomediated pro-inflammatory actions related to several factors, such as osteopontin, NF-κB and Toll-like receptors (141).

Various mechanisms of intracellular signaling have been elucidated, which contribute to the disease process in type 2 diabetes (Fig. 3).

i) In a previous study, osteopontin reduced the streptozotocin-induced nitric oxide levels in the islets through an arginine-glycine-aspartate (RGD)-dependent reduction of inducible nitric oxide synthase (iNOS) mRNA levels, resulting in improved glucose-stimulated insulin secretion (46).

ii) REG family proteins (small secretory proteins that promote proliferation and differentiation) are implicated in islet β-cell proliferation, survival and regeneration. The expression of pancreatitis-associated protein (PAP; REG3β) is highly induced in experimental diabetes and acute pancreatitis. It is anti-inflammatory in the liver and pancreatic acinars. Upon streptozotocin treatment, in contrast to wild-type littermates that became hyperglycemic in 3 days and lost 15% of their weight, RIP-I/REG3β mice were protected from hyperglycemia and weight loss. Islet-protective osteopontin and the acute responsive nuclear protein NUPR1 were induced by REG3β (142).

iii) Glucose-stimulated insulin secretion relies on Ca^2+^ influx into β-cells, which is modulated by the two-pore-domain K^+^ (K2P) channel, TALK-1, which modulates glucose-stimulated insulin secretion. A gain-of-function polymorphism in its encoding gene KCNK16 increases the risk for developing type 2 diabetes. Intracellular osteopontin (iOPN) is highly expressed in β-cells and is upregulated under pre-diabetic conditions to help maintain normal β-cell function. iOPN colocalized with and bound to TALK-1 channels in the pancreas. At physiological voltages, iOPN activated TALK-1 transcript variant 3 channels and K^+^ currents, which also hyperpolarized the resting membrane potential. Reducing β-cell iOPN decreased TALK-1 K^+^ currents and increased glucose-stimulated Ca^2+^ influx (143). Both osteopontin knockout and wild-type islets displayed synchronized Ca^2+^ oscillations, indicative of intact β-cell communication. Osteopontin knockout islets displayed higher intracellular Ca^2+^ concentrations when stimulated with glucose than wild-type islets and the initial dip upon elevated glucose concentrations (which is associated with Ca^2+^ uptake into the endoplasmic reticulum) was significantly lower in these islets (140).

iv) The pleiotropic effects of GIP on islet function involve osteopontin. mRNA expression of its receptor GIPR was decreased in human islets from carriers of the A allele of GIPR rs10423928 (associated with impaired glucose- and GIP-stimulated insulin secretion and a decrease in body-mass-index, lean body mass, and waist circumference) or in patients with type 2 diabetes (53).

The inhibition of sodium glucose cotransporter 2 (SGLT2) is a potential therapeutic strategy for treating diabetes. The SGLT2 inhibitor dapagliflozin ameliorated hyperglycemia, β-cell damage and albuminuria. Glomerular mesangial expansion and interstitial fibrosis were suppressed in a dose-dependent manner. Dapagliflozin treatment markedly decreased macrophage infiltration and the gene expression of inflammation and oxidative stress in the kidney. Osteopontin, TGF-α, MCP-1 and ICAM-1, were significantly increased in diabetes, but suppressed by dapagliflozin. Dapagliflozin ameliorates diabetic nephropathy by improving hyperglycemia along with inhibiting inflammation and oxidative stress (144).

*Cancer connection.* Diabetes and pancreatic cancer reciprocally increase the risk of developing each other.

i) Among diseases that are complications of pancreatic cancer, diabetes is the most common. Many cancer cases become evident, when patients are investigated for a worsening in glycemic control. Patients suffering from diabetes and obesity exhibit an increased expression of osteopontin, which may act as a critical regulator of insulin resistance and diabetes mellitus (131).

ii) Patients with diabetes have an elevated risk of developing pancreatic cancer (131). The levels of osteopontin were found to be higher in patients with ductal adenocarcinoma compared with patients with chronic type 2 diabetes (there were increased osteopontin levels in patients with chronic pancreatitis in comparison to those with type 2 diabetes and healthy controls) (80). In sera from patients with pancreatic lesions (comprising ductal adenocarcinoma and IPMN), the presence of diabetes and/or obesity was associated with complete disappearance of OPN-b and expression of only OPN-c. The splice variant OPN-c was significantly associated with diabetes and obesity (88).

Obesity is a risk and poor prognosis factor for ductal adenocarcinoma. In those patients, osteopontin expression in adipose tissues adjacent to adenocarcinoma tumor was higher than in non-obese patients. Obese adipocytes triggered aggressive transformation in ductal adenocarcinoma cells to induce progression and accelerate angiogenesis via osteopontin secretion (112). There is potential biochemical crosstalk between the metabolism of the adipose tissue (body mass) and bone marrow (peripheral trafficking of bone marrow-derived stem cells) in both healthy individuals and patients with obesity-associated cancer. Plasma osteopontin was increased in adenocarcinoma patients over healthy controls, but osteopontin alone was not a marker for obesity or progenitor cell trafficking. In adenocarcinoma patients, the osteonectin/osteopontin ratio significantly associated with the body mass index, as well as with the intensified systemic trafficking of mesenchymal and endothelial progenitor cells (82).

Pancreatic stellate cells contribute to tumor invasion, metastasis and chemoresistance. Osteopontin is highly expressed and secreted upon their activation (65). These cells support increased desmoplasia hyperplasia, a key mechanism by which obesity contributes to the progression of pancreatic cancer. Upon stimulation with TNF, IL-1/IL-2/IL-10, pancreatic stellate cells can be activated into cancer-associated fibroblast isoforms, such as iCAF and myCAF, which participate in the desmoplastic reaction to remodel the mesenchyme of pancreatic cancer (38).

## Discussion

*Summary.* Osteopontin has been understood as a key factor in the progression of about 30 types of malignancies (33,34). Specifically, its isoforms, entailing the splice variants -a, -b, and -c, are critical players in pancreatic cancer aggressiveness (35). Reported data have converged over the years on an understanding that osteopontin overexpression and aberrant splicing support the progression of cancers in the pancreatis. Furthermore, due to the cytokine functions of the full-length form OPN-a, the measurement of pan-osteopontin (total osteopontin) lacks specificity compared to measurement of the cancer-associated splice variants OPN-b and OPN-c. In support of the biomarker studies, functional insight has been gained, over several years of investigations, into the mechanisms of osteopontin-mediated cancer progression. Osteopontin facilitates the migration and invasion of cells (17). The full-length form and its cancer-specific splice variants OPN-b or OPN-c synergize in supporting the survival of deadherent cancer cells (14,30). These functions support metastasis, tropism, stemness and angiogenesis as outlined above.

Various precancerous conditions are capable of producing molecules of aggressiveness early in transformation, which reflects their cancer risk (34,145). In the pancreas, IPMN are bona fide precursor lesions of ductal adenocarcinoma. They often express and splice osteopontin, which serves as a biomarker for surveillance of their carcinogenesis risk (72). In pancreatic lesions (comprising ductal adenocarcinoma and IPMN), OPN-b and OPN-c were expressed in a substantial fraction of patients (88). Osteopontin aids the delineation of cancer from benign lesions (IPMN, pancreatitis) and healthy controls (91).

### Clinical implications

While several meta-analyses have corroborated the association of tumor-derived osteopontin and its variants with pancreatic cancer progression (33-35) and original reports on the abundance of osteopontin in cancerous tissue have had good agreement, initial analyses (with still maturing techniques) for the presence in surrounding tissue or for prognostic value had conflicting outcomes. The incongruences in the results by individual reports could reflect i) insufficient sensitivity or specificity when measuring total osteopontin; ii) structural variations in the protein combined with a lack of standardization of antibodies to be utilized for detection; iii) differences among pancreatic cancer subtypes; iv) tumor vs. stroma sources of osteopontin may also generate heterogeneity. Much more consistency has been achieved with osteopontin as a blood biomarker. It has potential for clinical applications when combined with organ-specific biomarkers (outlined in Table SIII).

Strong immune responses against pancreatic cancer have been hard to achieve. Osteopontin derived from tumor-infiltrating macrophages or in macrophage-fibroblast interactions has been associated with overall poor prognosis. Furthermore, despite the frequent presence of PD-L1, immune checkpoint blockers have had little efficacy. Osteopontin may compensate PD-L1 function to promote pancreatic cancer immune escape. However, pancreatic cancer may not have the levels of antigenicity required for prompting anti-tumor immunity. Whether the suppression of osteopontin in this setting could suffice for triggering an immune reaction to the pancreatic malignancy will require further research.

In non-transformed tissues, only the full-length form OPN-a is pertinent. In the healthy pancreas, osteopontin fulfills safeguarding functions, including islet survival and protection from hyperglycemia. However, its increased production is associated with pancreatitis, and there it may contribute to the inflammation.

The roles of osteopontin in diabetes are multi-faceted. In type 1 diabetes, the protein could be an autoantigen. However, it is also secreted during the disease and may have an ameliorating effect on the destructive inflammation. In type 2 diabetes, the β-cell protective functions seem to be in the foreground. Osteopontin is an islet protein and a pro-survival factor that is protective against both cytotoxicity and hyperglycemia, and may serve as an intrinsic feedback regulator of nitric oxide signaling in β-cells. Its temporal pattern of expression is inversely correlated with progression of insulitis and β-cell destruction.

*Connections.* The present systematic review has brought to the forefront several connections of osteopontin to other molecules and mechanisms.

Osteopontin avidly binds calcium and hydroxyapatite (18,146,147). This may be relevant for diseases of the pancreas.

i) Osteopontin is detectable in all cases of chronic calcifying pancreatitis and in approximately half of cases of chronic pancreatitis without stones. The molecule may play a critical role in stone formation (124). Autoimmune pancreatitis has the potential for calcification over a long-term clinical course. The incidence of osteopontin expression in centroacinar cells in chronic pancreatitis with calcification and in autoimmune pancreatitis was significantly greater than that in healthy controls (123).

ii) The ectopic mineralization of soft tissues is a typical response to systemic imbalance of various metabolic factors as well as tissue injury. Coxsackievirus B3 infection resulted in significant tissue injury, particularly in the pancreas and heart, involving substantial ectopic calcification. Infection induced upregulation of osteopontin, SPARC and collagen type I in the pancreas. α-lipoic acid diminished the tissue damage, subsequently ameliorating ectopic calcification via the suppression of osteogenic signals (148).

iii) Calcification is very rare in ductal adenocarcinomas, but sometimes focal dystrophic calcification arises. Psammoma bodies contain osteopontin, and their development may be facilitated by the high calcium-binding capacity of osteopontin (75,76).

The regulation of nitric oxide by osteopontin has long been known (149-152):

i) Pro-inflammatory cytokines and macrophage-derived IL-1β, TNF-α and nitric oxide perturb insulin secretion from transformed β-cells and whole islets. Osteopontin regulates nitric oxide production via inhibition of iNOS transcription. It dose-dependently improved islet cell-derived, glucose-stimulated insulin secretion and inhibited IL-1β-induced nitric oxide production in an arginine-glycine-aspartate (RGD)-dependent manner (120).

ii) The addition of osteopontin to diabetic islets significantly improved their glucose-stimulated insulin secretion. Osteopontin significantly reduced the streptozotocin-induced nitric oxide levels in the islets through an RGD-dependent reduction of iNOS mRNA levels (46).

The PPAR-γ agonist rosiglitazone is administered to improve glycemic control in adults with type 2 diabetes. It is typically prescribed as a monotherapy or in combination with metformin or sulfonylureas. In addition, the agent may be a treatment choice in acute pancreatitis and in post-transplant organ damage by cyclosporine A. In mice with acute pancreatitis fed a high-fat diet, the drug increased survival and hastened recovery from inflammation. This was associated with significantly lower circulating and pancreas-associated levels of osteopontin (122). The long-term administration of the immunosuppressant cyclosporine A post-transplantation causes hypoxic injury to the kidneys. Mediators include osteopontin. Rosiglitazone had a protective effect against pancreatic and renal injury caused by cyclosporine A. Pro-inflammatory and pro-fibrotic molecules, including osteopontin, also decreased under treatment (127).

*Conclusion.* The prognosis of pancreatic cancer is poor. This is due to the fact that, to a large extent, it is typically detected at advanced stages. Insights into its mechanisms of progression may lead to improved early detection and better late-stage treatments. Osteopontin variants, once sufficiently elucidated and delineated from non-cancerous diseases of the pancreas, are prominent candidate markers for this goal. The cancer-specific variant forms also represent candidate drug targets.

## Supplementary Material

PubMed-listed publications by year. The y-axis indicates the number of hits per calendar year. In the stacked bars, the lower set (dotted) shows articles in PubMed with the key phrase ‘pancreas cancer OR pancreatic cancer AND osteopontin’, the upper panel (hatched) displays articles in PubMed with the key phrase ‘osteopontin AND pancreas NOT cancer’.

PRISMA flow chart. The standard chart for identification of studies via databases and registers is displayed with the applicable numbers (153). Note that the reference citation pertains to the reference list in the main manuscript.

Fibroblast-macrophage interactions in ductal adenocarcinoma. Recruitment and reprogramming of fibroblasts and macrophages may generate an immunosuppressive environment by inhibiting cytotoxic T-cell functions.

Synopsis of the pertinent literature.

Patient studies.

Cancer markers used in conjunction with Osteopontin.

Blood biomarker osteopontin.

Osteopontin in pancreatic cancer immune responses.

Osteopontin in diabetes.

## Data Availability

The data generated in the present study may be requested from the corresponding author.

## References

[1] Zhao H, Chen Q, Alam A, Cui J, Suen KC, Soo AP, Eguchi S, Gu J, Ma D (2018). The role of osteopontin in the progression of solid organ tumour. Cell Death Dis.

[2] Chen L, Huan X, Xiao GH, Yu WH, Li TF, Gao XD, Zhang YC (2022). Osteopontin and its downstream carcinogenic molecules: Regulatory mechanisms and prognostic value in cancer progression. Neoplasma.

[3] Liu L, Niu K, Yang Z, Song J, Wei D, Zhang R, Tao K (2024). Osteopontin: An indispensable component in common liver, pancreatic, and biliary related disease. J Cancer Res Clin Oncol.

[4] Weber GF, Ashkar S (2000). Stress response genes: The genes that make cancer metastasize. J Mol Med (Berl).

[5] Weber GF (2008). Molecular mechanisms of metastasis. Cancer Lett.

[6] Senger DR, Wirth DF, Hynes RO (1979). Transformed mammalian cells secrete specific proteins and phosphoproteins. Cell.

[7] Senger DR, Wirth DF, Hynes RO (1980). Transformation-specific secreted phosophoproteins. Nature.

[8] Oldberg A, Franzen A, Heinegard D (1986). Cloning and sequence analysis of rat bone sialoprotein (osteopontin) cDNA reveals an Arg-Gly-Asp cell-binding sequence. Proc Natl Acad Sci USA.

[9] Craig AM, Nemir M, Mukherjee BB, Chambers AF, Denhardt DT (1988). Identification of the major phosphoprotein secreted by many rodent cell lines as 2ar/osteopontin: Enhanced expression in H-ras-transformed 3T3 cells. Biochem Biophys Res Commun.

[10] Senger DR, Perruzzi CA, Papadopoulos A, Tenen DG (1989). Purification of a human milk protein closely similar to tumor-secreted phosphoproteins and osteopontin. Biochim Biophys Acta.

[11] Senger DR, Perruzzi CA, Papadopoulos A (1989). Elevated expression of secreted phosphoprotein I (osteopontin, 2ar) as a consequence of neoplastic transformation. Anticancer Res.

[12] Miyauchi A, Alvarez J, Greenfield EM, Teti A, Grano M, Colucci S, Zambonin-Zallone A, Ross FP, Teitelbaum SL, Cheresh D (1993). Binding of osteopontin to the osteoclast integrin alpha v beta 3. Osteoporos Int.

[13] Weber GF, Ashkar S, Glimcher MJ, Cantor H (1996). Receptor-ligand interaction between CD44 and osteopontin (Eta-1). Science.

[14] He B, Mirza M, Weber GF (2006). An osteopontin splice variant induces anchorage independence in human breast cancer cells. Oncogene.

[15] Brown LF, Berse B, Van de Water L, Papadopoulos-Sergiou A, Perruzzi CA, Manseau EJ, Dvorak HF, Senger DR (1992). Expression and distribution of osteopontin in human tissues: Widespread association with luminal epithelial surfaces. Mol Biol Cell.

[16] Rudzki Z, Jothy S (1997). CD44 and the adhesion of neoplastic cells. Mol Pathol.

[17] Weber GF, Zawaideh S, Hikita S, Kumar VA, Cantor H, Ashkar S (2002). Phosphorylation-dependent interaction of osteopontin with its receptors regulates macrophage migration and activation. J Leukoc Biol.

[18] Prince CW (1989). Secondary structure predictions for rat osteopontin. Connect Tissue Res.

[19] Kurzbach D, Schwarz TC, Platzer G, Hofler S, Hinderberger D, Konrat R (2014). Compensatory adaptations of structural dynamics in an intrinsically disordered protein complex. Angew Chem Int Ed Engl.

[20] Kurzbach D, Canet E, Flamm AG, Jhajharia A, Weber EM, Konrat R, Bodenhausen G (2017). Investigation of intrinsically disordered proteins through exchange with hyperpolarized water. Angew Chem Int Ed Engl.

[21] Reinholt FP, Hultenby K, Oldberg A, Heinegard D (1990). Osteopontin-a possible anchor of osteoclasts to bone. Proc Natl Acad Sci USA.

[22] Fedarko NS, Fohr B, Robey PG, Young MF, Fisher LW (2000). Factor H binding to bone sialoprotein and osteopontin enables tumor cell evasion of complement-mediated attack. J Biol Chem.

[23] Chen Y, Gialeli C, Shen J, Duner P, Walse B, Duelli A, Caing-Carlsson R, Blom AM, Zibert JR, Nilsson AH (2024). Identification of an osteopontin-derived peptide that binds neuropilin-1 and activates vascular repair responses and angiogenesis. Pharmacol Res.

[24] Raineri D, Dianzani C, Cappellano G, Maione F, Baldanzi G, Iacobucci I, Clemente N, Baldone G, Boggio E, Gigliotti CL (2020). Osteopontin binds ICOSL promoting tumor metastasis. Commun Biol.

[25] Mark MP, Butler WT, Prince CW, Finkelman RD, Ruch JV (1988). Developmental expression of 44-kDa bone phosphoprotein (osteopontin) and bone gamma-carboxyglutamic acid (Gla)-containing protein (osteocalcin) in calcifying tissues of rat. Differentiation.

[26] Nagata T, Todescan R, Goldberg HA, Zhang Q, Sodek J (1989). Sulphation of secreted phosphoprotein I (SPPI, osteopontin) is associated with mineralized tissue formation. Biochem Biophys Res Commun.

[27] Hirota S, Ito A, Nagoshi J, Takeda M, Kurata A, Takatsuka Y, Kohri K, Nomura S, Kitamura Y (1995). Expression of bone matrix protein messenger ribonucleic acids in human breast cancers. Possible involvement of osteopontin in development of calcifying foci. Lab Invest.

[28] Bellahcene A, Castronovo V (1995). Increased expression of osteonectin and osteopontin, two bone matrix proteins, in human breast cancer. Am J Pathol.

[29] Yasui T, Fujita K, Sasaki S, Sato M, Sugimoto M, Hirota S, Kitamura Y, Nomura S, Kohri K (1999). Expression of bone matrix proteins in urolithiasis model rats. Urol Res.

[30] Fnu G, Weber GF (2023). Osteopontin induces mitochondrial biogenesis in deadherent cancer cells. Oncotarget.

[31] Briones-Orta MA, Avendano-Vazquez SE, Aparicio-Bautista DI, Coombes JD, Weber GF, Syn WK (2017). Osteopontin splice variants and polymorphisms in cancer progression and prognosis. Biochim Biophys Acta Rev Cancer.

[32] Shinohara ML, Kim HJ, Kim JH, Garcia VA, Cantor H (2008). Alternative translation of osteopontin generates intracellular and secreted isoforms that mediate distinct biological activities in dendritic cells. Proc Natl Acad Sci USA.

[33] Weber GF, Lett GS, Haubein NC (2010). Osteopontin is a marker for cancer aggressiveness and patient survival. Br J Cancer.

[34] Weber GF, Lett GS, Haubein NC (2011). Categorical meta-analysis of Osteopontin as a clinical cancer marker. Oncol Rep.

[35] An Y, Fnu G, Xie C, Weber GF (2023). Meta-analysis of osteopontin splice variants in cancer. BMC Cancer.

[36] Weber GF (2018). The phylogeny of Osteopontin-analysis of the protein sequence. Int J Mol Sci.

[37] Fisher LW, Torchia DA, Fohr B, Young MF, Fedarko NS (2001). Flexible structures of SIBLING proteins, bone sialoprotein, and osteopontin. Biochem Biophys Res Commun.

[38] Wang Q, Wang H, Ding Y, Wan M, Xu M (2022). The role of adipokines in pancreatic cancer. Front Oncol.

[39] Kilic G, Wang J, Sosa-Pineda B (2006). Osteopontin is a novel marker of pancreatic ductal tissues and of undifferentiated pancreatic precursors in mice. Dev Dyn.

[40] Hendley AM, Rao AA, Leonhardt L, Ashe S, Smith JA, Giacometti S, Peng XL, Jiang H, Berrios DI, Pawlak M (2021). Single-cell transcriptome analysis defines heterogeneity of the murine pancreatic ductal tree. Elife.

[41] Ojeda ML, Nogales F, Muñoz Del Valle P, Díaz-Castro J, Murillo ML, Carreras O (2016). Metabolic syndrome and selenium in fetal programming: Gender differences. Food Funct.

[42] Koopmann J, Fedarko NS, Jain A, Maitra A, Iacobuzio-Donahue C, Rahman A, Hruban RH, Yeo CJ, Goggins M (2004). Evaluation of osteopontin as biomarker for pancreatic adenocarcinoma. Cancer Epidemiol Biomarkers Prev.

[43] Tsai WC, Lin CK, Yang YS, Chan DC, Gao HW, Chang FN, Jin JS (2013). The correlations of LMX1A and osteopontin expression to the clinicopathologic stages in pancreatic adenocarcinoma. Appl Immunohistochem Mol Morphol.

[44] Weber CE, Erşahin ÇH, Kuo PC, Mi Z (2016). Pancreatic cancer and osteopontin: The relationship remains unclear. Pancreas.

[45] Collins AL, Rock J, Malhotra L, Frankel WL, Bloomston M (2012). Osteopontin expression is associated with improved survival in patients with pancreatic adenocarcinoma. Ann Surg Oncol.

[46] Katakam AK, Chipitsyna G, Gong Q, Vancha AR, Gabbeta J, Arafat HA (2005). Streptozotocin (STZ) mediates acute upregulation of serum and pancreatic osteopontin (OPN): A novel islet-protective effect of OPN through inhibition of STZ-induced nitric oxide production. J Endocrinol.

[47] Franklin O, Billing O, Öhlund D, Berglund A, Herdenberg C, Wang W, Hellman U, Sund M (2019). Novel prognostic markers within the CD44-stromal ligand network in pancreatic cancer. J Pathol Clin Res.

[48] Kolb A, Kleeff J, Guweidhi A, Esposito I, Giese NA, Adwan H, Giese T, Büchler MW, Berger MR, Friess H (2005). Osteopontin influences the invasiveness of pancreatic cancer cells and is increased in neoplastic and inflammatory conditions. Cancer Biol Ther.

[49] Franklin O, Öhlund D, Lundin C, Öman M, Naredi P, Wang W, Sund M (2015). Combining conventional and stroma-derived tumour markers in pancreatic ductal adenocarcinoma. Cancer Biomark.

[50] Anborgh PH, Wilson SM, Tuck AB, Winquist E, Schmidt N, Hart R, Kon S, Maeda M, Uede T, Stitt LW, Chambers AF (2009). New dual monoclonal ELISA for measuring plasma osteopontin as a biomarker associated with survival in prostate cancer: Clinical validation and comparison of multiple ELISAs. Clin Chem.

[51] Seiron P, Stenwall A, Hedin A, Granlund L, Esguerra JLS, Volkov P, Renström E, Korsgren O, Lundberg M, Skog O (2021). Transcriptional analysis of islets of Langerhans from organ donors of different ages. PLoS One.

[52] Kalnytska O, Qvist P, Kunz S, Conrad T, Willnow TE, Schmidt V (2024). SORCS2 activity in pancreatic α-cells safeguards insulin granule formation and release from glucose-stressed β-cells. iScience.

[53] Lyssenko V, Eliasson L, Kotova O, Pilgaard K, Wierup N, Salehi A, Wendt A, Jonsson A, De Marinis YZ, Berglund LM (2011). Pleiotropic effects of GIP on islet function involve osteopontin. Diabetes.

[54] Sedivy R, Peters K, Klöppel G (2005). Osteopontin expression in ductal adenocarcinomas and undifferentiated carcinomas of the pancreas. Virchows Arch.

[55] Wu W, Yang H, Wang Z, Zhang Z, Lu X, Yang W, Xu X, Jiang Y, Li Y, Fan X

[56] Chipitsyna G, Gong Q, Anandanadesan R, Alnajar A, Batra SK, Wittel UA, Cullen DM, Akhter MP, Denhardt DT, Yeo CJ, Arafat HA (2009). Induction of osteopontin expression by nicotine and cigarette smoke in the pancreas and pancreatic ductal adenocarcinoma cells. Int J Cancer.

[57] Tu Y, Chen C, Fan G (2019). Association between the expression of secreted phosphoprotein-related genes and prognosis of human cancer. BMC Cancer.

[58] Fiorino S, Visani M, Masetti M, Acquaviva G, Tallini G, De Leo A, Fornelli A, Ragazzi M, Vasuri F, Grifoni D (2020). Periostin, tenascin, osteopontin isoforms in long- and non-long survival patients with pancreatic cancer: A pilot study. Mol Biol Rep.

[59] Sullivan J, Blair L, Alnajar A, Aziz T, Ng CY, Chipitsyna G, Gong Q, Witkiewicz A, Weber GF, Denhardt DT (2009). Expression of a prometastatic splice variant of osteopontin, OPNC, in human pancreatic ductal adenocarcinoma. Surgery.

[60] Sullivan J, Blair L, Alnajar A, Aziz T, Chipitsyna G, Gong Q, Yeo CJ, Arafat HA (2011). Expression and regulation of nicotine receptor and osteopontin isoforms in human pancreatic ductal adenocarcinoma. Histol Histopathol.

[61] Van Heek NT, Maitra A, Koopmann J, Fedarko N, Jain A, Rahman A, Iacobuzio-Donahue CA, Adsay V, Ashfaq R, Yeo CJ (2004). Gene expression profiling identifies markers of ampullary adenocarcinoma. Cancer Biol Ther.

[62] Bloomston M, Ellison EC, Muscarella P, Al-Saif O, Martin EW, Melvin WS, Frankel WL (2007). Stromal osteonectin overexpression is associated with poor outcome in patients with ampullary cancer. Ann Surg Oncol.

[63] Hsu HP, Shan YS, Lai MD, Lin PW (2010). Osteopontin-positive infiltrating tumor-associated macrophages in bulky ampullary cancer predict survival. Cancer Biol Ther.

[64] Sedivy R, Kalipciyan M, Mazal PR, Wolf B, Wrba F, Karner-Hanusch J, Mühlbacher F, Mader RM (2005). Osteoclast-like giant cell tumor in mucinous cystadenocarcinoma of the pancreas: An immunohistochemical and molecular analysis. Cancer Detect Prev.

[65] Cao J, Li J, Sun L, Qin T, Xiao Y, Chen K, Qian W, Duan W, Lei J, Ma J (2019). Hypoxia-driven paracrine osteopontin/integrin αvβ3 signaling promotes pancreatic cancer cell epithelial-mesenchymal transition and cancer stem cell-like properties by modulating forkhead box protein M1. Mol Oncol.

[66] Lazar M, Sullivan J, Chipitsyna G, Gong Q, Ng CY, Salem AF, Aziz T, Witkiewicz A, Denhardt DT, Yeo CJ, Arafat HA (2010). Involvement of osteopontin in the matrix-degrading and proangiogenic changes mediated by nicotine in pancreatic cancer cells. J Gastrointest Surg.

[67] Lazar M, Sullivan J, Chipitsyna G, Aziz T, Salem AF, Gong Q, Witkiewicz A, Denhardt DT, Yeo CJ, Arafat HA (2010). Induction of monocyte chemoattractant protein-1 by nicotine in pancreatic ductal adenocarcinoma cells: Role of osteopontin. Surgery.

[68] Saxena S, Gandhi A, Lim PW, Relles D, Sarosiek K, Kang C, Chipitsyna G, Sendecki J, Yeo CJ, Arafat HA (2013). RAN GTPase and osteopontin in pancreatic cancer. Pancreat Disord Ther.

[69] Deng L, Ren J, Liu D, Li H, Yang G, Wang K, Song Y, Su H (2025). Ran drives pancreatic cancer metastasis by activating the osteopontin-PI3K/AKT-androgen receptor signaling cascade. Toxicol Appl Pharmacol.

[70] Grudzinska E, Szmigiel P, Majewska K, Mrowiec S, Czuba ZP (2025). Pancreatic cancer and benign pancreatic cystic lesions: Differences in cytokines, growth factors, and immunological markers concentrations in serum and cystic fluid. Cancers (Basel).

[71] Goluba K, Parfejevs V, Rostoka E, Jekabsons K, Blake I, Neimane A, Ule AA, Rimsa R, Vangravs R, Pcolkins A, Riekstina U (2024). Personalized PDAC chip with functional endothelial barrier for tumour biomarker detection: A platform for precision medicine applications. Mater Today Bio.

[72] Li J, Wei T, Ma K, Zhang J, Lu J, Zhao J, Huang J, Zeng T, Xie Y, Liang Y (2024). Single-cell RNA sequencing highlights epithelial and microenvironmental heterogeneity in malignant progression of pancreatic ductal adenocarcinoma. Cancer Lett.

[73] Siddiqui AA, Jones E, Andrade D, Shah A, Kowalski TE, Loren DE, Chipitsyna G, Arafat HA (2014). Osteopontin splice variant as a potential marker for metastatic disease in pancreatic adenocarcinoma. J Gastroenterol Hepatol.

[74] Coppola D, Szabo M, Boulware D, Muraca P, Alsarraj M, Chambers AF, Yeatman TJ (2004). Correlation of osteopontin protein expression and pathological stage across a wide variety of tumor histologies. Clin Cancer Res.

[75] Ohike N, Sato M, Kawahara M, Ohyama S, Morohoshi T (2008). Ductal adenocarcinoma of the pancreas with psammomatous calcification. Report of a case. JOP.

[76] Takayama S, Maeda T, Nishihara M, Kanazawa A, Chong HS, Oka H, Hirota S, Ishikawa O (2015). A case of intraductal tubulopapillary neoplasm of pancreas with severe calcification, a potential pitfall in diagnostic imaging. Pathol Int.

[77] Rao AD, Liu Y, von Eyben R, Hsu CC, Hu C, Rosati LM, Parekh A, Ng K, Hacker-Prietz A, Zheng L (2018). Multiplex proximity ligation assay to identify potential prognostic biomarkers for improved survival in locally advanced pancreatic cancer patients treated with stereotactic body radiation therapy. Int J Radiat Oncol Biol Phys.

[78] Kaleağasıoğlu F, Berger MR (2014). SIBLINGs and SPARC families: Their emerging roles in pancreatic cancer. World J Gastroenterol.

[79] Fredriksson S, Horecka J, Brustugun OT, Schlingemann J, Koong AC, Tibshirani R, Davis RW (2008). Multiplexed proximity ligation assays to profile putative plasma biomarkers relevant to pancreatic and ovarian cancer. Clin Chem.

[80] Rychlíková J, Vecka M, Jáchymová M, Macášek J, Hrabák P, Zeman M, Vávrová L, Řoupal J, Krechler T, Ák A (2016). Osteopontin as a discriminating marker for pancreatic cancer and chronic pancreatitis. Cancer Biomark.

[81] Li H, Lan L, Chen H, Zaw Thin M, Ps H, Nelson JK, Evans IM, Ruiz EJ, Cheng R, Tran L (2025). SPP1 is required for maintaining mesenchymal cell fate in pancreatic cancer. Nature.

[82] Blogowski W, Dolegowska K, Deskur A, Dolegowska B, Starzyńska T (2015). An attempt to evaluate selected aspects of ‘Bone-Fat Axis’ Function in healthy individuals and patients with pancreatic cancer. Medicine (Baltimore).

[83] Melen-Mucha G, Niedziela A, Mucha S, Motylewska E, Lawnicka H, Komorowski J, Stepien H (2012). Elevated peripheral blood plasma concentrations of tie-2 and angiopoietin 2 in patients with neuroendocrine tumors. Int J Mol Sci.

[84] Li JJ, Li HY, Gu F (2014). Diagnostic significance of serum osteopontin level for pancreatic cancer: A meta-analysis. Genet Test Mol Biomarkers.

[85] Koopmann J, Rosenzweig CN, Zhang Z, Canto MI, Brown DA, Hunter M, Yeo C, Chan DW, Breit SN, Goggins M (2006). Serum markers in patients with resectable pancreatic adenocarcinoma: Macrophage inhibitory cytokine 1 versus CA19-9. Clin Cancer Res.

[86] Chen R, Crispin DA, Pan S, Hawley S, McIntosh MW, May D, Anton-Culver H, Ziogas A, Bronner MP, Brentnall TA (2010). Pilot study of blood biomarker candidates for detection of pancreatic cancer. Pancreas.

[87] Hartung F, Weber GF (2013). RNA blood levels of osteopontin splice variants are cancer markers. Springerplus.

[88] Sarosiek K, Jones E, Chipitsyna G, Al-Zoubi M, Kang C, Saxena S, Gandhi AV, Sendiky J, Yeo CJ, Arafat HA (2015). Osteopontin (OPN) isoforms, diabetes, obesity, and cancer; what is one got to do with the other? A new role for OPN. J Gastrointest Surg.

[89] Rogers CD, Fukushima N, Sato N, Shi C, Prasad N, Hustinx SR, Matsubayashi H, Canto M, Eshleman JR, Hruban RH, Goggins M (2006). Differentiating pancreatic lesions by microarray and QPCR analysis of pancreatic juice RNAs. Cancer Biol Ther.

[90] Poruk KE, Firpo MA, Scaife CL, Adler DG, Emerson LL, Boucher KM, Mulvihill SJ (2013). Serum osteopontin and tissue inhibitor of metalloproteinase 1 as diagnostic and prognostic biomarkers for pancreatic adenocarcinoma. Pancreas.

[91] Song J, Sokoll LJ, Pasay JJ, Rubin AL, Li H, Bach DM, Chan DW, Zhang Z (2019). Identification of serum biomarker panels for the early detection of pancreatic cancer. Cancer Epidemiol Biomarkers Prev.

[92] Sekiguchi K, Matsuda A, Yamada M, Matsumoto S, Sakurazawa N, Kawano Y, Yamada T, Miyashita M, Yoshida H (2022). The utility of serum osteopontin levels for predicting postoperative complications after colorectal cancer surgery. Int J Clin Oncol.

[93] Jiménez-Fonseca P, Martín MN, Carmona-Bayonas A, Calvo A, Fernández-Mateos J, Redrado M, Capdevila J, Lago NM, Lacasta A, Muñarriz J (2018). Biomarkers and polymorphisms in pancreatic neuroendocrine tumors treated with sunitinib. Oncotarget.

[94] Melisi D, Garcia-Carbonero R, Macarulla T, Pezet D, Deplanque G, Fuchs M, Trojan J, Kozloff M, Simionato F, Cleverly A (2019). TGFβ receptor inhibitor galunisertib is linked to inflammation- and remodeling-related proteins in patients with pancreatic cancer. Cancer Chemother Pharmacol.

[95] Chen K, Wang Q, Liu X, Wang F, Ma Y, Zhang S, Shao Z, Yang Y, Tian X (2020). Single cell RNA-Seq identifies Immune-related prognostic model and key Signature-SPP1 in pancreatic ductal adenocarcinoma. Genes (Basel).

[96] Raymant M, Astuti Y, Alvaro-Espinosa L, Green D, Quaranta V, Bellomo G, Glenn M, Chandran-Gorner V, Palmer DH, Halloran C (2024). Macrophage-fibroblast JAK/STAT dependent crosstalk promotes liver metastatic outgrowth in pancreatic cancer. Nat Commun.

[97] Lu C, Liu Z, Klement JD, Yang D, Merting AD, Poschel D, Albers T, Waller JL, Shi H, Liu K (2021). WDR5-H3K4me3 epigenetic axis regulates OPN expression to compensate PD-L1 function to promote pancreatic cancer immune escape. J Immunother Cancer.

[98] Yang Y, Gong Y, Ding Y, Sun S, Bai R, Zhuo S, Zhang Z (2024). LINC01133 promotes pancreatic ductal adenocarcinoma epithelial-mesenchymal transition mediated by SPP1 through binding to Arp3. Cell Death Dis.

[99] Gaviraghi M, Tunici P, Valensin S, Rossi M, Giordano C, Magnoni L, Dandrea M, Montagna L, Ritelli R, Scarpa A, Bakker A (2011). Pancreatic cancer spheres are more than just aggregates of stem marker-positive cells. Biosci Rep.

[100] Nose K, Saito H, Kuroki T (1990). Isolation of a gene sequence induced later by tumor-promoting 12-O-tetradecanoylphorbol-13-acetate in mouse osteoblastic cells (MC3T3-E1) and expressed constitutively in ras-transformed cells. Cell Growth Differ.

[101] Pitarresi JR, Norgard RJ, Chiarella AM, Suzuki K, Bakir B, Sahu V, Li J, Zhao J, Marchand B, Wengyn MD (2021). PTHrP drives pancreatic cancer growth and metastasis and reveals a new therapeutic vulnerability. Cancer Discov.

[102] Vanova K, Boukalova S, Gbelcova H, Muchova L, Neuzil J, Gurlich R, Ruml T, Vitek L (2016). Heme oxygenase is not involved in the anti-proliferative effects of statins on pancreatic cancer cells. BMC Cancer.

[103] Adams CR, Htwe HH, Marsh T, Wang AL, Montoya ML, Subbaraj L, Tward AD, Bardeesy N, Perera RM (2019). Transcriptional control of subtype switching ensures adaptation and growth of pancreatic cancer. Elife.

[104] Nishimori H, Yasoshima T, Hata F, Denno R, Yanai Y, Nomura H, Tanaka H, Kamiguchi K, Sato N, Hirata K (2002). A novel nude mouse model of liver metastasis and peritoneal dissemination from the same human pancreatic cancer line. Pancreas.

[105] Ohno K, Nishimori H, Yasoshima T, Kamiguchi K, Hata F, Fukui R, Okuya K, Kimura Y, Denno R, Kon S (2010). Inhibition of osteopontin reduces liver metastasis of human pancreatic cancer xenografts injected into the spleen in a mouse model. Surg Today.

[106] Zhivkova-Galunska M, Adwan H, Eyol E, Kleeff J, Kolb A, Bergmann F, Berger MR (2010). Osteopontin but not osteonectin favors the metastatic growth of pancreatic cancer cell lines. Cancer Biol Ther.

[107] Delany AM (2010). Matricellular proteins osteopontin and osteonectin/SPARC in pancreatic carcinoma. Cancer Biol Ther.

[108] Eyol E, Murtaga A, Zhivkova-Galunska M, Georges R, Zepp M, Djandji D, Kleeff J, Berger MR, Adwan H (2012). Few genes are associated with the capability of pancreatic ductal adenocarcinoma cells to grow in the liver of nude rats. Oncol Rep.

[109] Yang MC, Wang HC, Hou YC, Tung HL, Chiu TJ, Shan YS (2015). Blockade of autophagy reduces pancreatic cancer stem cell activity and potentiates the tumoricidal effect of gemcitabine. Mol Cancer.

[110] Ouyang L, Sun MM, Zhou PS, Ren YW, Liu XY, Wei WY, Song ZS, Lu K, Yang LX (2024). LncRNA FOXD1-AS1 regulates pancreatic cancer stem cell properties and 5-FU resistance by regulating the miR-570-3p/SPP1 axis as a ceRNA. Cancer Cell Int.

[111] Nallasamy P, Nimmakayala RK, Karmakar S, Leon F, Seshacharyulu P, Lakshmanan I, Rachagani S, Mallya K, Zhang C, Ly QP (2021). Pancreatic tumor microenvironment factor promotes cancer stemness via SPP1-CD44 Axis. Gastroenterology.

[112] Fukusada S, Shimura T, Natsume M, Nishigaki R, Okuda Y, Iwasaki H, Sugimura N, Kitagawa M, Katano T, Tanaka M (2024). Osteopontin secreted from obese adipocytes enhances angiogenesis and promotes progression of pancreatic ductal adenocarcinoma in obesity. Cell Oncol (Dordr).

[113] Liu Y, Fu Y, Liu T, Wang J, Zhang Z, Shi Y, Cao Z, Yang G, Chen H, Luo W (2025). SPP1+ venous endothelial cells and CRABP2+ tumor cells contribute to favorable body and tail pancreatic ductal adenocarcinoma tumor microenvironment. Mol Carcinog.

[114] Li X, Tjalkens RB, Shrestha R, Safe S (2019). Structure-dependent activation of gene expression by bis-indole and quinoline-derived activators of nuclear receptor 4A2. Chem Biol Drug Des.

[115] Nolen BM, Brand RE, Prosser D, Velikokhatnaya L, Allen PJ, Zeh HJ, Grizzle WE, Huang Y, Lomakin A, Lokshin AE (2014). Prediagnostic serum biomarkers as early detection tools for pancreatic cancer in a large prospective cohort study. PLoS One.

[116] Hill RL, Yadav D, Hart PA, Whitcomb DC, McQuerry KJ, Karnik KN, Stello KM, Conwell DL, Rao M (2025). Circulating molecular drivers of bone remodeling in pancreatitis. Clin Transl Gastroenterol.

[117] Rao C, Bush N, Rana SS, Sharma RK, Rana S, Jeyashree K, Gupta R (2022). Plasma osteopontin levels as an early predictor of mortality in acute pancreatitis: A preliminary study. Pancreas.

[118] Sward P, Bertilsson S, Struglics A, Kalaitzakis E (2018). Serum osteopontin is associated with organ failure in patients with acute pancreatitis. Pancreas.

[119] Aggarwal S, Xie MH, Maruoka M, Foster J, Gurney AL (2001). Acinar cells of the pancreas are a target of interleukin-22. J Interferon Cytokine Res.

[120] Arafat HA, Katakam AK, Chipitsyna G, Gong Q, Vancha AR, Gabbeta J, Dafoe DC (2007). Osteopontin protects the islets and beta-cells from interleukin-1 beta-mediated cytotoxicity through negative feedback regulation of nitric oxide. Endocrinology.

[121] Llewellyn HP, Vaidya VS, Wang Z, Peng Q, Hyde C, Potter D, Wang J, Zong Q, Arat S, Martin M (2021). Evaluating the sensitivity and specificity of promising circulating biomarkers to diagnose liver injury in humans. Toxicol Sci.

[122] Pini M, Rhodes DH, Castellanos KJ, Cabay RJ, Grady EF, Fantuzzi G (2012). Rosiglitazone improves survival and hastens recovery from pancreatic inflammation in obese mice. PLoS One.

[123] Takada H, Nakazawa T, Ohara H, Ando T, Hayashi K, Naito I, Okumura F, Tanaka H, Yamada T, Takahashi S, Joh T (2009). Role of osteopontin in calcification in autoimmune pancreatitis. Dig Dis Sci.

[124] Nakamura M, Oka M, Iizuka N, Kawauchi S, Gondo T, Ueno T, Tangoku A (2002). Osteopontin expression in chronic pancreatitis. Pancreas.

[125] Cheng Y, Li Y, Scherer N, Grundner-Culemann F, Lehtimäki T, Mishra BH, Raitakari OT, Nauck M, Eckardt KU, Sekula P (2022). Genetics of osteopontin in patients with chronic kidney disease: The German Chronic Kidney Disease study. PLoS Genet.

[126] Togashi Y, Miyamoto Y (2013). Urinary cystatin C as a biomarker for diabetic nephropathy and its immunohistochemical localization in kidney in Zucker diabetic fatty (ZDF) rats. Exp Toxicol Pathol.

[127] Chung BH, Li C, Sun BK, Lim SW, Ahn KO, Yang JH, Choi YH, Yoon KH, Sugawara A, Ito S (2005). Rosiglitazone protects against cyclosporine-induced pancreatic and renal injury in rats. Am J Transplant.

[128] Meerwein C, Korom S, Arni S, Inci I, Weder W, Jungraithmayr W (2011). The effect of low-dose Continuous Erythropoietin receptor activator in an experimental model of acute Cyclosporine A induced renal injury. Eur J Pharmacol.

[129] Glazunova AM, Arutyunova MS, Tarasov EV, Nikankina LV, Ilyin AV, Shamkhalova MS, Shestakova MV, Moysyuk YG (2016). Evaluation of markers for renal graft dysfunction in patients with type 1 diabetes mellitus after kidney transplantation and simultaneous pancreas-kidney transplantation. Ter Arkh.

[130] Gong Q, Chipitsyna G, Gray CF, Anandanadesan R, Arafat HA (2009). Expression and regulation of osteopontin in type 1 diabetes. Islets.

[131] Ito M, Makino N, Matsuda A, Ikeda Y, Kakizaki Y, Saito Y, Ueno Y, Kawata S (2017). High glucose accelerates cell proliferation and increases the secretion and mRNA expression of osteopontin in human pancreatic duct epithelial cells. Int J Mol Sci.

[132] Melanitou E (2020). Investigation of type 1 diabetes in NOD mice knockout for the osteopontin gene. Gene.

[133] Garbossa SG, Folli F (2017). Vitamin D, sub-inflammation and insulin resistance. A window on a potential role for the interaction between bone and glucose metabolism. Rev Endocr Metab Disord.

[134] Daniele G, Winnier D, Mari A, Bruder J, Fourcaudot M, Pengou Z, Hansis-Diarte A, Jenkinson C, Tripathy D, Folli F (2018). The potential role of the osteopontin-osteocalcin-osteoprotegerin triad in the pathogenesis of prediabetes in humans. Acta Diabetol.

[135] Fierabracci A, Biro PA, Yiangou Y, Mennuni C, Luzzago A, Ludvigsson J, Cortese R, Bottazzo GF (1999). Osteopontin is an autoantigen of the somatostatin cells in human islets: Identification by screening random peptide libraries with sera of patients with insulin-dependent diabetes mellitus. Vaccine.

[136] Aspord C, Rome S, Thivolet C (2004). Early events in islets and pancreatic lymph nodes in autoimmune diabetes. J Autoimmun.

[137] Arafat HA, Lada E, Katakam AK, Amin N (2006). Osteopontin deficiency impacts the pancreatic TH1/TH2 cytokine profile following multiple low dose streptozotocin-induced diabetes. Exp Clin Endocrinol Diabetes.

[138] Safley SA, Kenyon NS, Berman DM, Barber GF, Willman M, Duncanson S, Iwakoshi N, Holdcraft R, Gazda L, Thompson P (2018). Microencapsulated adult porcine islets transplanted intraperitoneally in streptozotocin-diabetic non-human primates. Xenotransplantation.

[139] Cai M, Bompada P, Salehi A, Acosta JR, Prasad RB, Atac D, Laakso M, Groop L, De Marinis Y (2018). Role of osteopontin and its regulation in pancreatic islet. Biochem Biophys Res Commun.

[140] Wendt A, Mollet IG, Knutsson A, Bolmgren VS, Hultgardh-Nilsson A, Gomez MF, Eliasson L (2017). Osteopontin affects insulin vesicle localization and Ca2+ homeostasis in pancreatic beta cells from female mice. PLoS One.

[141] Sommese L, Zullo A, Mancini FP, Fabbricini R, Soricelli A, Napoli C (2017). Clinical relevance of epigenetics in the onset and management of type 2 diabetes mellitus. Epigenetics.

[142] Xiong X, Wang X, Li B, Chowdhury S, Lu Y, Srikant CB, Ning G, Liu JL (2011). Pancreatic islet-specific overexpression of Reg3β protein induced the expression of pro-islet genes and protected the mice against streptozotocin-induced diabetes mellitus. Am J Physiol Endocrinol Metab.

[143] Dickerson MT, Vierra NC, Milian SC, Dadi PK, Jacobson DA (2017). Osteopontin activates the diabetes-associated potassium channel TALK-1 in pancreatic β-cells. PLoS One.

[144] Terami N, Ogawa D, Tachibana H, Hatanaka T, Wada J, Nakatsuka A, Eguchi J, Horiguchi CS, Nishii N, Yamada H (2014). Long-term treatment with the sodium glucose cotransporter 2 inhibitor, dapagliflozin, ameliorates glucose homeostasis and diabetic nephropathy in db/db mice. PLoS One.

[145] Walaszek K, Lower EE, Ziolkowski P, Weber GF (2018). Breast cancer risk in premalignant lesions: Osteopontin splice variants indicate prognosis. Br J Cancer.

[146] Shiraga H, Min W, VanDusen WJ, Clayman MD, Miner D, Terrell CH, Sherbotie JR, Foreman JW, Przysiecki C, Neilson EG (1992). Inhibition of calcium oxalate crystal growth in vitro by uropontin: Another member of the aspartic acid-rich protein superfamily. Proc Natl Acad Sci USA.

[147] Gorski JP, Shimizu K (1988). Isolation of new phosphorylated glycoprotein from mineralized phase of bone that exhibits limited homology to adhesive protein osteopontin. J Biol Chem.

[148] Kim HS, Shin HI, Lim HS, Lee TY, Lee K, Jeong D (2013). α-Lipoic acid attenuates coxsackievirus B3-induced ectopic calcification in heart, pancreas, and lung. Biochem Biophys Res Commun.

[149] Hwang SM, Lopez CA, Heck DE, Gardner CR, Laskin DL, Laskin JD, Denhardt DT (1994). Osteopontin inhibits induction of nitric oxide synthase gene expression by inflammatory mediators in mouse kidney epithelial cells. J Biol Chem.

[150] Miyazaki Y, Tashiro T, Higuchi Y, Setoguchi M, Yamamoto S, Nagai H, Nasu M, Vassalli P (1995). Expression of osteopontin in a macrophage cell line and in transgenic mice with pulmonary fibrosis resulting from the lung expression of a tumor necrosis factor-alpha transgene. Ann N Y Acad Sci.

[151] Singh K, Balligand JL, Fischer TA, Smith TW, Kelly RA (1995). Glucocorticoids increase osteopontin expression in cardiac myocytes and microvascular endothelial cells. Role in regulation of inducible nitric oxide synthase. J Biol Chem.

[152] Rollo EE, Laskin DL, Denhardt DT (1996). Osteopontin inhibits nitric oxide production and cytotoxicity by activated RAW264.7 macrophages. J Leukoc Biol.

[153] Page MJ, McKenzie JE, Bossuyt PM, Boutron I, Hoffmann TC, Mulrow CD, Shamseer L, Tetzlaff JM, Akl EA, Brennan SE (2021). The PRISMA 2020 statement: An updated guideline for reporting systematic reviews. BMJ.

