# Supplementary material for: Osteopontin in pancreatic cancer: A systematic review
Source: Med Int (Lond). 2026 Mar 23;6(3):26. doi: 10.3892/mi.2026.310 (PMC13040131; doi:10.3892/mi.2026.310)

Figure S1. PubMed-listed publications by year. The y-axis indicates the number of hits per calendar year. In the stacked bars, the lower set (dotted) shows articles in PubMed with the key phrase ‘pancreas cancer OR pancreatic cancer AND osteopontin’, the upper panel (hatched) displays articles in PubMed with the key phrase ‘osteopontin AND pancreas NOT cancer’.

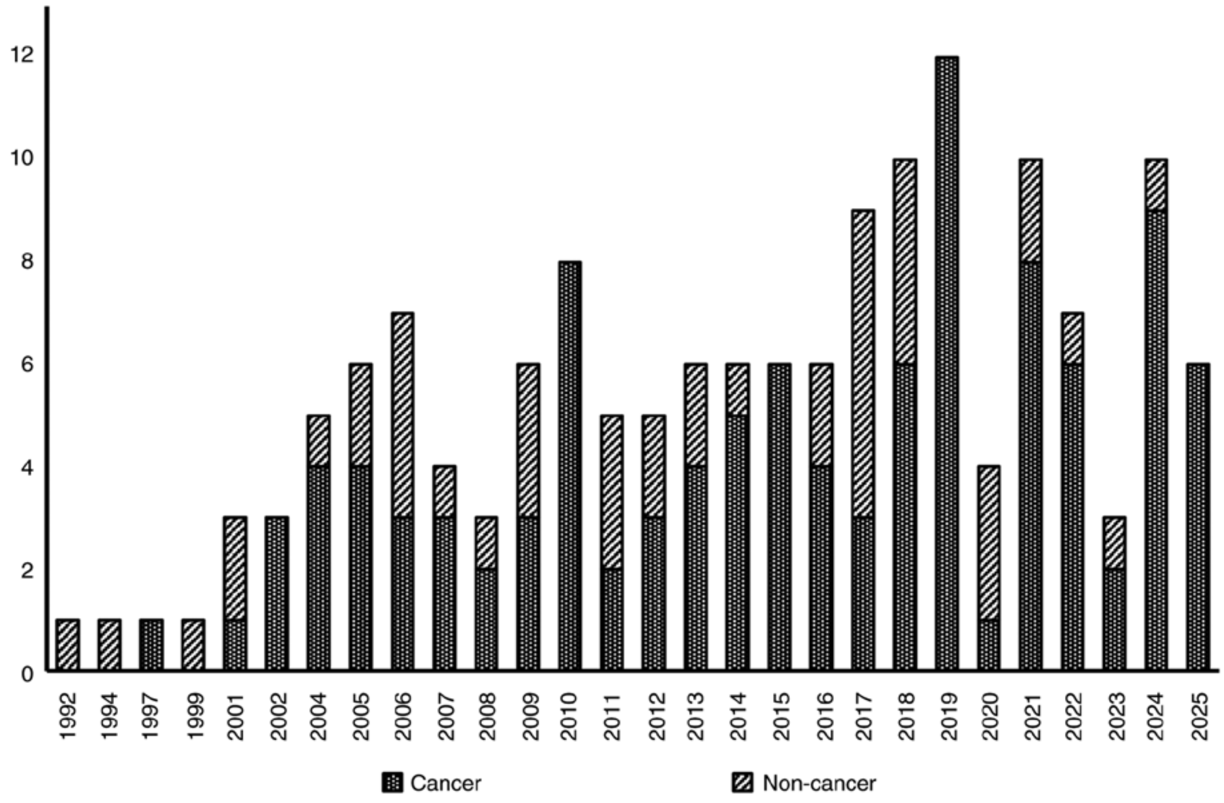

Figure S2. PRISMA flow chart. The standard chart for identification of studies via databases and registers is displayed with the applicable numbers (153). Note that the reference citation pertains to the reference list in the main manuscript.

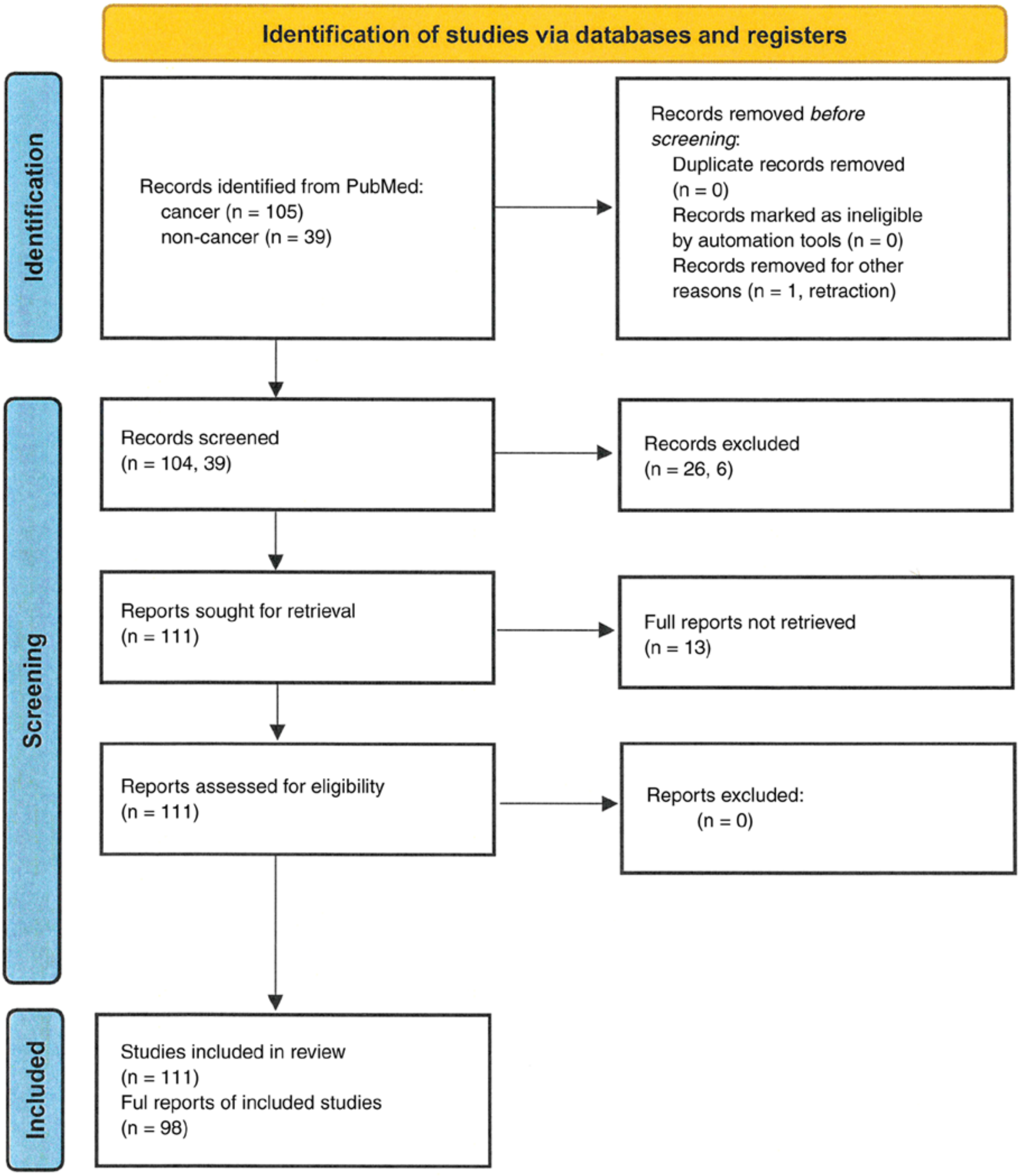

Figure S3. Fibroblast-macrophage interactions in ductal adenocarcinoma. Recruitment and reprogramming of fibroblasts and macrophages may generate an immunosuppressive environment by inhibiting cytotoxic T-cell functions.

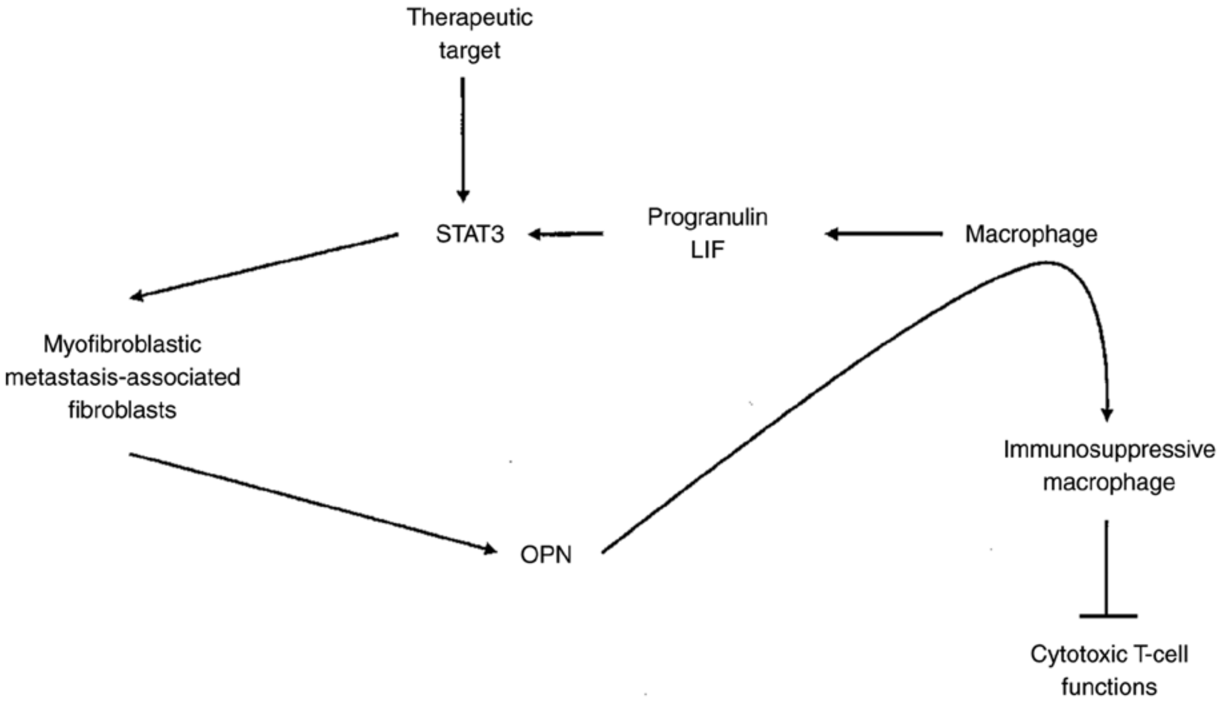

Supplement: PubMed-listed publications by year. The y-axis indicates the number of hits per calendar year. In the stacked bars, the lower set (dotted) shows articles in PubMed with the key phrase ‘pancreas cancer OR pancreatic cancer AND osteopontin’, the upper panel (hatched) displays articles in PubMed with t [file Supplementary_Data1.pdf]
