# Supplementary material for: Osteopontin in pancreatic cancer: A systematic review
Source: Med Int (Lond). 2026 Mar 23;6(3):26. doi: 10.3892/mi.2026.310 (PMC13040131; doi:10.3892/mi.2026.310)
Supplement: Synopsis of the pertinent literature. [file Supplementary_Data2.pdf]

Table S1. Synopsis of the pertinent literature.						
First author	OPN	Condition	Effect	Cells/biofluids	Species	(Refs.)[a]
Fierabracci	pan-OPN	Diabetes mellitus	Autoantigen	Somatostatin cells	Human	(135)
Aggarwal	pan-OPN	Pancreatitis	Induction, inflammation	Acinar cells	Mouse	(119)
Nishimori	pan-OPN	Metastatic tropism	Association with liver metastases	Human pancreas cancer sublines	Nude Mouse	(104)
Nakamura	pan-OPN	Chronic pancreatitis	Chronic calcifying pancreatitis, pancreatitis without stones, healthy pancreas	Acinar or ductal cells	Human	(124)
Coppola	pan-OPN	Cancer	Tissue marker	Epithelial cells	Human	(74)
Koopmann	pan-OPN	Adenocarcinoma	Expression and tumor stage (tissue, serum)	Epithelial cells, macrophages, serum	Human	(42)
Van Heek	pan-OPN	Ampullary adenocarcinoma	Differentiation from adenoma and duodenum	Epithelial cells, serum	Human	(61)
Aspord	pan-OPN	Autoimmune diabetes	Induction	Islets, pancreatic lymph nodes	Mouse	(136)
Sedivy	pan-OPN	Ductal adenocarcinoma, undifferentiated carcinoma	Differentiation between tumor types	Epithelial cells, macrophages	Human	(54)
Sedivy	pan-OPN	Mucinous cystadenocarcinoma, osteoclast-like giant cell tumor	Case study	Osteoclast-like giant cells	Human	(64)
Kolb	pan-OPN	Cancer progression	Invasiveness	Ductal adenocarcinoma	Human	(48)
Chung	pan-OPN	Cyclosporine injury	Rosiglitazone response	Pancreas tissue	Rat	(127)

Katakam	pan-OPN	Diabetes	Induction, protection from streptozotocin	Pancreas tissue, serum	Rat	(46)
Koopmann	pan-OPN	Resectable adenocarcinoma	Biomarker	Serum	Human	(85)
Kilic	pan-OPN	Morphogenesis, homeostasis	Cell migration, cell-cell interactions	Undifferentiated pancreatic precursors, pancreatic ductal tissues	Mouse	(39)
Rogers	pan-OPN	Cancer	Gene expression profile	Pancreatic juice	Human	(89)
Arafat	pan-OPN	Diabetes	Protection from streptozotocin	Pancreas tissue	Mouse	(137)
Bloomston	pan-OPN	Periampullary cancer	Relation to biomarker osteonectin	Cancer and stroma	Human	(62)
Arafat	pan-OPN	Inflammation	Protection of islet and beta cells	Beta cells	Rat	(120)
Fredriksson	pan-OPN	Cancer	Biomarker	Plasma	Human	(79)
Ohike	pan-OPN	Ductal adenocarcinoma, psammomatous calcification	calcification	Cancer cells	Human	(75)
Takada	pan-OPN	Autoimmune pancreatitis	Calcification	Pancreas tissue	Human, rat	(123)
Chipitsyna	pan-OPN	Ductal adenocarcinoma	Smoking etiology (SPP1 promoter)	Cancer cells	Human, rat	(56)
Gong	pan-OPN	Prediabetes	Expression	Islets, beta cells	Mouse	(130)
Sullivan	pan-OPN, OPN-c	Ductal adenocarcinoma	Smoking	Cancer cells	Human	(59)
Ohno	pan-OPN	Metastasis	Liver metastasis	Metastatic sublines	Human	(105)
Chen	pan-OPN	Cancer	Biomarker	Blood	Human	(86)

Hsu	pan-OPN	Ampullary adenocarcinoma	Metastasis	Tumor-associated macrophages	Human	(63)
Zhivkova-Galunska	pan-OPN	Ductal adenocarcinoma	Liver metastasis	Cancer cells	Nude rat	(106)
Lazar	pan-OPN, OPNc	Ductal adenocarcinoma	Smoking, inflammation	Cancer cells	Human	(67)
Lazar	pan-OPN, OPN	Ductal adenocarcinoma	Smoking, angiogenesis	Cancer cells	Human	(66)
Gaviraghi	pan-OPN	Stemness	Sphere formation, invasive capacity	Cell lines	Human	(99)
Xiong	pan-OPN	Diabetes	Protection from streptozotozin	Islets	Mouse	(142)
Sullivan	OPNa, OPNb, OPNc	Ductal adenocarcinoma	Smoking, metastasis	Cancer cells	Human	(60)
Lyssenko	pan-OPN	Diabetes	Mediation of GIP effects	Islets	Human, mouse, rat	(53)
Meerwein	pan-OPN	Post-transplantation diabetes	Protection from cyclosporin A	Pancreas tissue	rat	(128)
Melen-Mucha	pan-OPN	Neuroendocrine cancer	Biomarker	Plasma	Human	(83)
Pini	pan-OPN	Acute pancreatitis	Rosiglitazone-induced recovery	Blood	Mouse	(122)
Collins	pan-OPN	Adenocarcinoma	Survival	Cancer cells	Human	(45)
Eyol	pan-OPN	Ductal adenocarcinoma	Liver metastasis	Cancer cells	Human, rat	(108)
Kim	pan-OPN	Coxsackie virus infection	Ectopic calcification	Pancreas tissue	Mouse	(148)
Tsai	pan-OPN	Ductal adenocarcinoma	Grade, stage	Cancer cells	Human	(43)

Poruk	pan-OPN	Ductal adenocarcinoma	Biomarker	Serum	Human	(90)
Togashi	pan-OPN	Diabetes	Renal damage	Biomarker	Rat	(126)
Hartung	OPNa, OPNb, OPNc	Cancer	Biomarker	Blood	Human	(87)
Saxena	pan-OPN	Ductal adenocarcinoma	Perineural invasion, metastasis	Cancer cells	Human	(68)
Siddiqui	OPNa, OPNb, OPNc	Cancer, pancreatitis	Biomarker	Tissue	Human	(73)
Nolen	pan-OPN	Predisposition	Biomarker	Serum	Human	(115)
Terami	pan-OPN	Diabetic nephropathy	Glucose homeostasis, inflammation	Renal cortex	Mouse	(144)
Li	pan-OPN	Meta-analysis	Biomarker	Serum	Human	(84)
Franklin	pan-OPN	Ductal adenocarcinoma	Biomarker	Tumor tissue, plasma	Human	(49)
Sarosiek	OPNa, OPNb, OPNc	Pancreatic lesions	Biomarker	Serum, cell lines	Human	(88)
Takayama	pan-OPN	Intraductal tubulopapillary neoplasms	Biomarker	Tissue	Human	(76)
Blogowski	pan-OPN	Adenocarcinoma, obesity	Biomarker	Plasma	Human	(82)
Yang	pan-OPN	Cancer	Autophagy	Cancer stem cells	Human, Mouse	(109)
Vanova	pan-OPN	Treatment with statins	Invasion	Cancer cells	Human	(103)
Rychlíková	pan-OPN	Ductal adenocarcinoma, pancreatitis	Biomarker	Serum	Human	(80)

Ojeda	pan-OPN	Metabolic syndrome	Biomarker	Serum	Rat	(41)
Weber	pan-OPN	Ductal adenocarcinoma	Biomarker, progression	Cancer cells, cell lines	Human	(44)
Glazunova	pan-OPN	Diabetes, kidney transplantaiton	Biomarker (kidney function)	Serum	Human	(129)
Wendt	pan-OPN	Type 2 diabetes	Insulin vesicle localization, calcium homeostasis	Beta cells	Mouse	(140)
Ito	pan-OPN	Diabetes, obesity	Proliferation, induction of OPN	Cell lines	Human	(131)
Dickerson	iOPN	Diabetes	Potassium channel activation	Beta cells	Mouse	(143)
Cai	pan-OPN	Diabetes	Islet protection	Islets	Human, mouse	(139)
Rao	pan-OPN	Cancer: Chemotherapy, radiation	Biomarker	Serum	Human	(77)
Daniele	pan-OPN	Prediabates	Biomarker	Serum	Human	(134å
Swärd	pan-OPN	Acute pancreatitis	Inflammation, plasma glucose, organ failure	Serum	Human	(118)
Safley	pan-OPN	Diabetes	Hypoxia marker	Islets	Non-human primate	(138)
Jiménez-Fonseca	pan-OPN	Metastatic neuroendocrine cancer	Response to sunitinib	Serum	Human	(93)
Song	pan-OPN	Adenocarcinoma	Biomarker	Serum	Human	(91)
Cao	pan-OPN	Cancer	EMT, stemness	Tissue, cancer cell lines, stellate cells	Human	(65)
Franklin	pan-OPN	Cancer	Biomarker	Tissue	Human	(47)

Melisi	pan-OPN	Unresectable adenocarcinoma	Treatment response	Plasma	Human	(94)
Li	pan-OPN	Cancer	Gene expression under treatment	Cell lines	Human	(114)
Adams	pan-OPN	Ductal adenocarcinoma	Basal-like conversion	Cell lines, tissue	Human, mouse	(103)
Tu	pan-OPN	Ductal adenocarcinoma, intraepithelial neoplasia	Biomarker	Tissue	Human	(57)
Fiorino	pan-OPN, OPNa, OPNb, OPNc	Ductal adenocarcinoma	Survival	Tissue	Human	(58)
Melanitou	OPN alleles	Non-obese diabetes	Protection	*In vivo* knockout	Mouse	(132)
Llewellyn	pan-OPN	Pancreatitis	Biomarker	Serum	Human	(121)
Seiron	pan-OPN	Diabetes	Insulin production	Islets	Human	(51)
Hendley	pan-OPN	Development, pancreatitis	Regulation of EMT	Pancreatic duct cells	Human, mouse	(40)
Pitarresi	pan-OPN	Ductal adenocarcinoma	Induction by PTHrP and tumor progression	Cancer cells	Human	(101)
Lu	pan-OPN	Cancer	Immune escape	Cancer cells, myeloid-derived suppressor cells	Human, Mouse	(97)
Nallasamy	pan-OPN	Cancer stemness	Microenvironment	Cancer cells, fibroblasts	Human	(111)
Wu	pan-OPN	Ductal adenocarcinoma	Tumor progression	Cancer cells	Human	(55)
Cheng	pan-OPN	Chronic kidney disease	Pancreatic expression	Pancreas cells	Human	(125)
Rao		Acute pancreatitis	Biomarker	Plasma	Human	(117)

Wang	pan-OPN	Cancer	Adipokine effects on cancer	Cancer cells, leukocytes, mesenchymal stem cells	Human	(38)
Sekiguchi	pan-OPN	Hepatobiliary pancreatic cancer	Postoperative complications	Serum biomarker	Human	(92)
Chen	pan-OPN	Ductal adenocarcinoma	Overall survival	Malignant ductal cells	Human	(95)
Kalnytska	pan-OPN	Glucose stress	Ssecretion	Alpha cells, beta cells	Mouse	(52)
Fukusada	pan-OPN	Ductal adenocarcinoma, obesity	Tumor growth, angiogenesis	Cancer cells, adipocytes, endothelial cells	Human, mouse	(112)
Ouyang	pan-OPN	Cancer	Long non-coding RNA, stemness	Cancer stem cells, cell lines	Human	(110)
Li	pan-OPN	Intraductal papillary mucinous neoplasm	Biomarker	Macrophages, epithelial cells	Human	(72)
Raymant	pan-OPN	Ductal adenocarcinoma	Metastasis, immunosuppression	Fibroblasts, macrophages	Human, mouse	(96)
Yang	pan-OPN	Ductal adenocarcinoma	Epithelial-mesenchymal transition	Cancer cells, adjacent healthy cells	Human	(98)
Goluba	pan-OPN	Ductal adenocarcinoma	Biomarker, endothelial barrier	Tumor-derived organoids, endothelial cells	Human	(71)
Deng	pan-OPN	Cancer	Metastasis	Cancer cells	Human	(69)

[a]The reference citations pertain to the reference list in the main manuscript. The references that form the basis for the present systematic review are listed in chronological order. With bibliographic information, the osteopontin (OPN) forms under study, the conditions, effects observed, the cell types or biofluids studied, and the organism of focus are included.

Table SII. Patient studies.																	
Disease	Specimen	Analyte	Technique	Readout	No. of subjects	F	M	Age	panOPN	OPNa	OPNb	OPNc	Target	Stats	Comments	First author	(Refs.)[a]
Healthy	Tissue	mRNA	Gene expression profiling	Relative abundance	33								Aging		Upregulated expression with age	Seiron	(51)
Chronic kidney disease	Tissue	mRNA	GWAS		4,897			60 years (SD 12)	OPN and SPP1 in pancreas				Renal disease	H4: p12 = 0.85	Colocalization of the OPN association signal at SPP1 with expression in pancreas tissue	Cheng	(125)
Pancreatitis	Serum	Protein	Electro-chemiluminescence	log ng/ml	34				~ 1.1				Tissue injury		No correlation of OPN with ALT (r = -0.2086, p = 0 .0336)	Llewellyn	(121)
Liver damage	Serum	Protein	Electro-chemiluminescence	log ng/ml	104				~ 1.8								
Muscle damage	Serum	Protein	Electro-chemiluminescence	log ng/ml	74				~ 1.3								
Kidney damage	Serum	Protein	Electro-chemiluminescence	log ng/ml	40				~ 1.5								
Healthy	Serum	Protein	Electro-chemiluminescence	log ng/ml	135				1								
Chronic pancreatitis, calcifying	Tissue	mRNA	RT-PCR, ISH	Positivity	11				100% OPN+				Pancreatitis	P=0.0081	Acinar or ductal cells	Nakamura	(124)
Chronic pancreatitis, no stones	Tissue	mRNA	RT-PCR, ISH	Positivity	9				56% OPN+								
Healthy	Tissue	mRNA	RT-PCR, ISH	Positivity	5				0% OPN+								
Autoimmune pancreatitis	Tissue	Protein	Western blotting	Positive/ negative	12	2	10	64 years, range 44-78	10 centroacinar, 5 ductal					P<0.05 versus healthy		Takada	(123)

Chronic pancreatitis	Tissue	Protein	Western blotting	Positive/ negative	21	2	19	56 years, range 14-81	13 centroacinar, 7 ductal					P<0.05 versus healthy	11 chronic pancreatitis without calcification, ten chronic pancreatitis with calcification, 12 alcoholic, nine idiopathic		
healthy	Tissue	Protein	Western blotting	Positive/ negative	9	5	4	60 years, range 43-75	1 centroacinar, 0 ductal						four intraductal papillary mucinous neoplasms, two mucinous cystic tumors, two tumors of the ampulla of Vater, one insulinoma		
Acute pancreatitis, mild	Serum	Protein	ELISA	ng/ml	62				36 (27-47)					P=0.005	OPN positively correlated to APACHE II scores (rS = 0.34, P = 0.002), serum levels of IL-6 (rS = 0.60, P < 0.001), TNF-α (rS = 0.50,P < 0.001); no difference between alcohol-induced pancreatitis and others	Swärd	(118)
Acute pancreatitis, moderate to severe	Serum	Protein	ELISA	ng/ml	24				49 (35–78)						all patients (n = 86): 38 (30–52)		
Acute pancreatitis, organ failure	Serum	Protein	ELISA	ng/ml	13				77 (38–87)					p = 0.003; AUC(admission) 0.76 (95% CI, 0.60–9.93); AUC(12-24 hours) 0.83 (95% CI, 0.69–0.96)	independent predictors of organ failure upon admission: serum osteopontin (OR, 6.0; CI 1.2–30.8) and serum IL-6 (OR, 1.95; 95% CI, 1.09–3.51)	Swärd	(118)
Acute pancreatitis, no organ failure	Serum	Protein	ELISA	ng/ml	73				37 (27-47)								

Acute pancreatitis	Plasma	Protein	ELISA	ng/ml	50	19	31	36.6, std 11.48						significantly elevation for death versus survivors (28.5 [SD, 7.41] ng/mL vs 18.4 [SD, 8.73] ng/mL; P = 0.032). ROC curve 0.823 (95% CI 0.569–1.077; P = 0.035)	no difference with/without persistent organ failure (20.9 [SD, 8.53] ng/mL)(18.5 [SD, 8.99] ng/mL) (P = 0.35); respiratory failure (21.08 ng/mL, P = 0.826), renal failure (22.07 ng/mL, P = 0.958), shock (20.5 ng/mL, P = 0.944); with/without pancreatic necrosis (18.7 [SD, 9.09] ng/mL)(17.7 [SD, 7.93] ng/mL)(P = 0.73)	Rao	(117)
Normal glucose tolerance	Serum	Protein	Milliplex Map	ng/ml	43				3.3±0.2				Diabetes	P<0.05		Daniele	(134)
Impaired glucose regulation	Serum	Protein	Milliplex Map	ng/ml	79				5.3±0.5								
Impaired fasting glucose	Serum	Protein	Milliplex Map	ng/ml	27				4.5±0.3								
Impaired glucose tolerance	Serum	Protein	Milliplex Map	ng/ml	18				6.3±0.5								
Impaired fasting glucose, impaired glucose tolerance	Serum	Protein	Milliplex Map	ng/ml	34				5.4±0.5								

Glucose tolerance subgroups	Serum	Protein	Milliplex Map	mgl										Positive correlation with fasting glucose (r = 0.27; p = 0.002), 2-hours plasma glucose (r = 0.24; p = 0.004), HbA1c (r = 0.31; p < 0.001),fasting plasma insulin (r = 0.17; p = 0.04); negative correlation with OGIS (r = − 0.22; p = 0.01), TGD/SSPI (r = − 0.28; p = 0.02)		Daniele	(134)
Pancreatic lesions, comorbidities	Serum	mRNA	RT-qPCR	Relative abundance	40				110					p < 0.05 from healthy, p > 0.05 between groups		Sarosiek	(88)
Pancreatic lesions, no comorbidities	Serum	mRNA	RT-qPCR	Relative abundance	50				130								
Diabetes, kidney trransplant					41									Significant increase over control		Glazunova	(129)
Diabetes, pancreas-kidney transplant					20									Significant increase over control			
Diabetes without nephropathy					15												

Pancreatic cancer, smoker	Tumor	mRNA	RT-qPCR	Relative abundance	29				high=21, medium=4, low=2, none=2				cancer risk / predisposition		present in 87% of invasive cancer lesions	Sullivan	(59)
Pancreatic cancer, non-smoker	Tumor	mRNA	RT-qPCR	Relative abundance	11				high=4, medium=3, low=1, none=2								
Pancreatic cancer, all prediagnosis	Serum	Protein	multiplexed bead-based immunoassay						CA 19-9, OPG, OPN: sensitivity = 30.4% specificity = 84.7% area-under-the-curve = 0.547						no significant difference from CA19-9 alone	Nolen	(115)
Pancreatic cancer, 1-12 months to diagnosis	Serum	Protein	Multiplexed bead-based immunoassay						CA 19-9, OPG, OPN: sensitivity = 37.9% specificity = 84.7% area-under-the-curve = 0.596								
Pancreatic cancer, 12-35 months to diagnosis	Serum	Protein	Multiplexed bead-based immunoassay						CA 19-9, OPG, OPN: sensitivity = 22.2% specificity = 84.7% area-under-the-curve = 0.494								
Pancreatic lesion (PDA or IPMN)	Serum	mRNA	RT-qPCR	Relative abundance	50					0% positive	48% positive	37% positive				Sarosiek	(88)
Pancreatic lesion (PDA or IPMN)	Serum	mRNA	RT-qPCR	Relative abundance	17					0% positive	0% positive	100% positive		OPNb: OR=0.01 (CI 0.001-1.2) p=0.603; OPNc:			

														OR=56.85 (CI 0.67-infinite) p=0.074			
Pancreatic lesion (PDA or IPMN)	Serum	mRNA	RT-qPCR	Relative abundance	6					0% positive	0% positive	100% positive		OPNb: OR=0.1 (CI 0.03-0.36) p=0.000; OPNc: OR=7.06 (CI 1.97-25.3) p=0.003	OPNc association with diabetes and obesity (OR=7.06 [95 % CI 1.97–23.3]; p=0.003)		
Pancreatic lesion (PDA or IPMN)	Serum	mRNA	RT-qPCR	Relative abundance	17					0% positive	0% positive	100% positive		OPNb: OR=0.26 (CI 0.7-0.96) p=0.435; OPNc: OR=2.73 (CI 0.73-10.26) p=0.676			
No lesion	Serum	mRNA	RT-qPCR	Relative abundance	29					0% positive	0% positive	0% positive					
Intraductal tubulopapillary neoplasms (ITPN)	Tissue	Protein	immunohistochemistry	Path score	1				Positive						psammoma body-type and non psammoma body-type calcifications were positive	Takayama	(76)
Adenocarcinoma	Plasma	Protein	ELISA	ng/ml	35	23	13	60±7	~ 3.5					p < 0.05	osteonectin/osteopontin ratio associated with BMI (r = 0.52, p < 0.05) and intensified systemic trafficking of mesenchymal (r = 0.49, P < 0.05) and endothelial progenitor cells (r = 0.55, P = 0.04)	Blogowski	(82)

Healthy	Plasma	Protein	ELISA	ng/ml	35	22	13	56±11	~ 2						no correlation with circulating stem cell populations		
Adenocarcinoma, lean	Plasma	Protein	ELISA	ng/ml										no significance		Blogowski	(82)
Adenocarcinoma, obese	Plasma	Protein	ELISA	ng/ml													
Healthy, lean	Plasma	Protein	ELISA	ng/ml										p = 0.72		Blogowski	(82)
Healthy, obese	Plasma	Protein	ELISA	ng/ml													
IPMN	Serum	Protein	Multiplex proximity ligation assay		63			64	low					P < 0.001 (AUC 0.80, [0.72–0.86])		Song	(91)
Pancreatitis	Serum	Protein	Multiplex proximity ligation assay		68			51	high								
Cancer	Tissue	Protein	IHC	Allred score	16				0 = 2, 1 to 3 = 1, 4 to 6 = 4, 7 to 8 = 9				association with cancer		13 positive (81%)	Coppola	(74)
Healthy	Tissue	Protein	IHC	Allred score											negative or minimal stain		
Pancreatic cancer	Serum	Protein	ELISA	ng/ml	50				mean = 623, median = 604, std = 327						also correlation to MIC-1, CA19-9, HIP, TIMP-1	Koopmann	(85)
Chronic pancreatitis	Serum	Protein	ELISA	ng/ml	50				mean = 408, median = 366, std = 270								
Normal	Serum	Protein	ELISA	ng/ml	50				mean = 321, median = 258, std = 213								

Pancreatic cancer	Pancreatic juice	mRNA	microarray	Relative abundance	7			range = 43–79	5.1-fold overexpression (compared to combined "non-cancer" controls)					p=0.023		Rogers	(89)
chronic pancreatitis	Pancreatic juice	mRNA	microarray	Relative abundance	5			range = 25–78									
benign disease	Pancreatic juice	mRNA	microarray	Relative abundance	4			range = 41–56									
Pancreatic cancer	Plasma	Protein	Multiplexed Proximity Ligation Assay	Relative abundance	18				mean = 215 (interquartile range 185-270, range 70-785)						also correlation with ADAM-8, CA-125, CA 19-9, carboxypeptidase A1, CEA, connective tissue growth factor, EGFR, E-CAM, Her2, galectin-1, insulin-like growth factor 2, IL-1 , IL-7, mesothelin, macrophage migration inhibitory factor, secretory leukocyte peptidase inhibitor, TNF, VEGF, chitinase 3–like 1	Fredriksson	(79)
Normal	Plasma	Protein	Multiplexed Proximity Ligation Assay	Relative abundance	19				mean = 100 (interquartile range 90-125, range 70-235)								
Pancreatic cancer	Serum	Protein	ELISA	Relative abundance	20				1.98±0.1					p = 0.01 vs. healthy, p = 0.001 vs. all controls	OPN vs. healthy: AUC = 0.99, sensitivity = 95%, specificity = 80%	Chen	(86)

Pancreatitis	Serum	Protein	ELISA	Relative abundance	20				1.45±0.18						OPN vs. all controls: AUC = 0.76, sensitivity = 0%, specificity = 28%		
Healthy	Serum	Protein	ELISA	Relative abundance	5												
Pancreatic cancer	Tumor	Protein	IHC	Path score	15				OPN+ 93%							Hsu	(63)
Ampullary cancer	Tumor	Protein	IHC	Path score	100				OPN+ 33%								
Pancreatic cancer	Blood	mRNA	qRT-PCR	Relative abundance	7	4	3	65.3 (range 43-85)		0.0059826676525 4608 +/- 0.0069916484575 1333 (mean +/- std)	0.033240228289 5052 +/- 0.067970108372 6482 (mean +/- std)	1.5949466 2998 674 +/- 3.6134804 8381 218 (mean +/- std)		OPNb, OPNc up $p < 0.05$		Hartung	(87)
Healthy	Blood	mRNA	qRT-PCR	Relative abundance	74	62	7	51.1 (range 18-85)		0.0144114343945 356 +/- 0.0383633413388 854 (mean +/- std)	0.008913489234 40223 +/- 0.018892089875 2173 (mean +/- std)	0.1533331 5701 5514 +/- 0.3875266 9959 3947 (mean +/- std)					
Pancreatic cancer					491									patients elevated over healthy controls (SMD=3.58, 95% CI=2.42-	11 clinical case-control studies	Li	(84)

														4.74, p<0.001)			
Healthy					481									ethnicity stratified analysis(Caucasians: SMD=2.62, 95% CI=1.33-3.91, p<0.001; Asians: SMD=4.54, 95% CI=2.80-6.27, p<0.001)			
Cancer	Tissue	Protein	IHC	Path score	75	32	43		~ 7.5 ; strong expression in stromal cells adjacent to tumor cells					P < 0.01	OPN and FOXM1 significantly upregulated in pancreatic cancer tissues and associated with poor clinical outcome	Cao	(65)
Healthy	Tissue	Protein	IHC	Path score	25				~ 1.5 ; rare staining events in the stroma and pancreatic ductal cells								
Cancer	Tissue	Protein	IHC	Path score	67				positive 62 (93%)						healthy acinar cells strongly CD44s + in cell membranes weakly CD44v6 + in cytoplasm. cancer cells membranous CD44v6 + , stromal	Franklin	(47)

															cells in cancer CD44s +		
Cancer	Tissue	mRNA	Microarray						overexpression, not significant					threshold: p = 1E-4, fold change = 2, gene ranking = 10%	Oncomine , 1 study	Tu	(57)
Normal	Tissue	mRNA	Microarray														
Carcinoma	Tissue	Protein	IHC	Path score	5				~75% positive, level 4.8					percent p = 0.0284, level p < 0.0001		Lu	(97)
Healthy	Tissue	Protein	IHC	Path score	5				~50% positive, level 1.7								
Carcinoma	Tissue	mRNA	Gene expression profiling	Relative abundance	167									p < 0.0001	TCGA	Lu	(97)
Healthy	Tissue	mRNA	Gene expression profiling	Relative abundance	182												
Carcinoma	Serum	Protein	ELISA	ng/ml	20				~ 30					p = 0.0071		Lu	(97)
Healthy	Serum	Protein	ELISA	ng/ml	20				~ 17								
Adenocarcinoma	Tissue	mRNA	ISH	Path score	14				0						in tumor-infiltrating macrophages in 8 of 14 (57%)	Koopmann	(42)
Ductal adenocarcinoma	Tumor	Protein	IHC	Path score	15				OPN - = 5, OPN + = 7, OPN ++ = 1, OPN +++ = 2						no OPN correlation to E-cadherin, β-Catenin	Sedivy	(54)
Undifferentiated carcinoma	Tumor	Protein	IHC	Path score	10				OPN - = 3, OPN + = 3, OPN ++ = 2, OPN +++ = 2								

Ductal adenocarcinoma	Tumor	mRNA	RT-PCR	transcripts/µl	23				5053±907					p < 0.05	PDAC and pancreatitis not significant	Kolb	(48)
Chronic pancreatitis	Tissue	mRNA	RT-PCR	transcripts/µl	22				3653±632					p = 0.08			
Healthy pancreas	Tissue	mRNA	RT-PCR	transcripts/µl	20				2259±441								
Ductal adenocarcinoma	Serum	Protein	ELISA	ng/ml	70				UICC stage I/II 120 ± 12 ng/ml UICC stage III/IV 126 ± 20 ng/ml					p < 0.05	1.6-fold over healthy	Kolb	(48)
Chronic pancreatitis	Serum	Protein	ELISA	ng/ml	12				143 ± 24					p < 0.01	1.9-fold over healthy		
Healthy pancreas	Serum	Protein	ELISA	ng/ml	20				76 ± 11								
ductal adenocarcinoma, diffuse psammomatous calcification	Tumor	Protein	IHC	Path score	1			83	OPN positive = 1						OPN positivity in psammoma bodies	Ohike	(75)
															also correlation to CK7, MUC1, CA 19-9, CEA, p53, MUC2, chromogranin A		
Invasive ductal adenocarcinoma	Tissue	mRNA	RT-qPCR	Relative abundance	25				800							Chipitsyna	(56)
Premalignant lesion					11				200								
Non-malignant tissue									200								
Invasive ductal adenocarcinoma	Tissue	Protein	IHC	Positivity											intense, localized to the membrane and cytoplasm of the tumor cells	Chipitsyna	(56)
Premalignant lesion															in the transforming ducts		
Non-malignant tissue															focally present and mostly on the apical surface of the ductal epithelium		

Resected adenocarcinoma	Tumor	Protein	IHC	Path score	245				74% positive							Collins	(45)
Healthy	Tumor	Protein	IHC	Path score	12				58% positive								
Ductal adenocarcinoma	Serum	Protein	ELISA	ng/ml	86				77.6±67.3 ng/ml					from pancreatitis p ≤ 0.0001; from healthy p < 0.0001	iterative classification tree analyses with TIMP-1 and CA19-9 improved sensitivity (0.87), specificity (0.91)	Poruk	(90)
Resectable adenocarcinoma	Serum	Protein	ELISA	ng/ml	45				62.4±50.9 ng/ml						subset of 86 cancers		
Chronic pancreatitis	Serum	Protein	ELISA	ng/ml	48				41.8±29.8 ng/ml					pancreatitis versus healthy p = 0.774			
Healthy	Serum	Protein	ELISA	ng/ml	86				39.5±32.8 ng/ml						cancer: increased levels (p < 0.0001) in older patients		
Ductal adenocarcinoma	Tumor, fine needle aspirate	mRNA	RT-qPCR	Relative abundance	40	17	23	68.1 (45–89)		98% positive, 186±36 transcripts/µl	60% positive, 179.6±41 transcripts/µl	25% positive, 192.1 ±21 transcripts/µl		OPNa, OPNb p < 0.05	no differences by demographics, clinical presentation: OPNc associated with metastasis (OR=9.3 [95% CI=1.6–52.9]), smoking (OR=13.5 [95% CI=1.5–120.8]; p=0.009)	Siddiqui	(73)
Chronic pancreatitis	Tissue, fine needle aspirate	mRNA	RT-qPCR	Relative abundance	6	2	4	57.8 (44–71)		50% positive, 147 ± 32 transcripts/µl	17% positive, 173 transcripts/µl	0% positive					

Ductal adenocarcinoma	Serum	Protein	Multiplexed bead-based immunoassay		343	180	163	median 68, range 29–92	CA 19-9, OPG, OPN: sensitivity = 82.4% specificity = 95% area-under-the-curve = 0.954						2.3% stage 1, 20% stage 2, 10% stage 3, 25% stage 4, 39% unknown	Nolen	(115)
healthy	Serum	Protein	Multiplexed bead-based immunoassay		227	139	88	median 56, range 18–87									
Invasive ductal adenocarcinoma	Tissue	mRNA	RT-qPCR	Relative abundance	51	25	26	67.5 years						elevated ($p < 0.0002$) in tumor over adjacent tissue	significant correlation between ln(OPN) and RAN for all tissues	Saxena	(68)
benign lesions	Tissue	mRNA	RT-qPCR	Relative abundance	7	6	1	59.9 years						elevated ($p = 0.0054$) in cancer over benign lesions	IPMN, n=4; cystadenoma, n=3		
adjacent nonmalignant tissue	Tissue	mRNA	RT-qPCR	Relative abundance	22	13	9	68.1 years									
Ductal adenocarcinoma	Tissue	Protein	Immuno-fluorescence	Path score	8				weak intracellular staining in all cancer cells					stroma stained positive but weaker in intensity than the cancer cells		Franklin	(49)
healthy	Tissue	Protein	Immuno-fluorescence	Path score	4				central areas of acini exclusively stained								
Ductal adenocarcinoma	Serum	mRNA	RT-qPCR	Relative abundance	58	32	26	65.82		0% positive	19% positive	43% positive				Sarosiek	(88)
IPMN	Serum	mRNA	RT-qPCR	Relative abundance	32	21	14	66.34		0% positive	7% positive	25% positive					

Ductal adenocarcinoma	Serum	Protein	ELISA	ng/ml	64				~130					PDAC versus CP (P < 0.001), PDAC versus T2DM (P < 0.001), PDAC versus healthy (P < 0.001)	correlation with bilirubin (r = 0.244, P < 0.001), AST (r = 0.269, P < 0.001), GGT (r = 0.450, P < 0.001), ALP (r = 0.557, P < 0.001), CRP (r = 0.412, P < 0.001), CEA (r = 0.487, P < 0.001), CA 19-9 (r = 0.445, P < 0.001), CA 72-4 (r = 0.193, P < 0.01); negative correlation with albumin (r = −0.559, P < 0.001), HDL-cholesterol (r = −0.295,P < 0.001)	Rychlíková	(80)
Chronic pancreatitis	Serum	Protein	ELISA	ng/ml	71				~75					CP versus T2DM (P < 0.001), CP versus healthy (P < 0.001)	at cut-off of 102 ng/ml, differentiation PDAC from CP: sensitivity 64%, specificity 81% (P < 0.001). No correlation with CP stage		
Type 2 diabetes	Serum	Protein	ELISA	ng/ml	67				~50						no correlation with duration of diabetes (cut-off 3 years)		
healthy	Serum	Protein	ELISA	ng/ml	48				~55								
Adenocarcinoma	Tissue	Protein	IHC	Path score	57				20 negative, 21 weak, 4 moderate low, 2 moderate high, 10 strong						benign and malignant pancreatic ductal cells displayed cytoplasmic and luminal staining; no nuclear staining	Weber	(44)
Healthy	Tissue	Protein	IHC	Path score	10				2 negative, 5 weak, 1 moderate low, 1 moderate								

									high, 1 strong								
Adenocarcinoma, stage 1	Tissue	Protein	IHC	Path score	12										neither the quantity nor the intensity of staining correlated with any of the available pathological variables	Weber	(44)
Adenocarcinoma, stage 2	Tissue	Protein	IHC	Path score	38												
Adenocarcinoma stage 3	Tissue	Protein	IHC	Path score	5												
Adenocarcinoma stage 4	Tissue	Protein	IHC	Path score	2												
Adenocarcinoma	Serum	Protein	Multiplex proximity ligation assay		188									from healthy P < 0.0001 (AUC 0.90, [0.86–0.94]); from benign P < 0.0001	6-plex immunoassay of OPN, MIA, CEACAM-1, MIC-1, SPON1, HSP27	Song	(91)
Pancreatitis, IPMN	Serum	Protein	Multiplex proximity ligation assay		131									AUC(OPN) = 0.80 vs. AUC(CA19-9) = 0.70, P < 0.01	outperformed CA19-9 in separating IPMN from chronic pancreatitis		
Healthy	Serum	Protein	Multiplex proximity ligation assay		89									benign from healthy (AUC = 0.80, [0.74–0.85])			
Adenocarcinoma	Tissue	mRNA (TCGA)	Gene expression profile	GEPIA	179				~ 9					P < 0.05		Cao	(65)

healthy	Tissue	mRNA (TCGA)	Gene expression profile	GEPIA	171				~ 6								
Dustal adenocarcinoma	Tissue	mRNA	Microarray		78				6.619-fold					p = 4.26 x 10E-11	over normal	Tu	(57)
Intraepithelial neoplasia	Tissue	mRNA	Microarray		38				1.827-fold					p = 3.10 x 10E-5			
Ductal adenocarcinoma	Tissue	mRNA	qRT-PCR	Relative abundance	21				9 positive						2 (9.5%) expressed OPNa, 3 (14.3%) OPN-c, 2 (9.5%) OPNb and OPNc, 3 (14.3%) had OPNa and OPNb and OPNc	Fiorino	(58)
Ductal adenocarcinoma	Tissue	Protein	IHC	Path score	50	18	32	28-81 (mean 61.64)	70% strongly positive							Wu	(55)
surrounding healthy tissue	Tissue	Protein	IHC	Path score	50	18	32	28-81 (mean 61.64)	32% strongly positive								
Ductal adenocarcinoma	Tissue	mRNA	TCGA	TPM					~ 500					$p < 0.05$		Chen	(95)
Surrounding tissue	Tissue	mRNA	TCGA	TPM					~ 100								
Ductal adenocarcinoma	Tissue	mRNA	TCGA	Relative abundance	179				~ 9					$p < 0.05$		Chen	(95)
Surrounding tissue	Tissue	mRNA	TCGA	Relative abundance	171				~ 6								
Ductal adenocarcinoma	Tissue	RNA	TCGA_PAAD	Relative abundance	179				~ 9					$p < 0.05$		Li	(72)
Healthy tissue	Tissue	RNA	TCGA_PAAD and GTEx	Relative abundance	171				~ 6								
Neuroendocrine cancer	Plasma	Protein	ELISA	ng/ml	2										also other gut neuroendocrine cancers	Melen-Mucha	(83)
Healthy	Plasma	Protein	ELISA	ng/ml	16												
Ampullary neoplasm	Tumor	mRNA	ISH	Path score	50				70% positive, 15 negative, 22 weak, 13 strong						also comparison to Osteonectin	Bloomston	(62)

Chronic pancreatitis	Tissue	mRNA	ISH	Path score	12				75% positive, 3 negative, 9 weak, 0 strong								
Normal pancreas	Tissue	mRNA	ISH	Path score	17				82% positive, 3 negative, 14 weak, 0 strong								
Ampullary adenocarcinoma	Tumor	mRNA	microarray	Relative abundance	5				fold change = 26.95					p = 0.00197		Van Heek	(61)
Normal duodenum	Tissue	mRNA	microarray	Relative abundance	10												
Ampullary adenocarcinoma	Tumor	mRNA	ISH	Path score	54				100% positive						infiltrating macrophages	Van Heek	(61)
Ampullary adenocarcinoma	Serum	Protein	ELISA	ng/ml	28				905±268						no correlation between OPN and preoperative serum bilirubin or TNM stage	Van Heek	(61)
Ampullary adenoma	Serum	Protein	ELISA	ng/ml	6				867±160								
Nonmalignant periampullary disease	Serum	Protein	ELISA	ng/ml					327±196								
Normal duodenum	Serum	Protein	ELISA	ng/ml	22				204±65								
Osteoclast-like giant cell tumor in mucinous cystadeno-carcinoma	Tumor	Protein	IHC	Path score	1			44	OPN = ++						also correlation with her2/neu, mismatch repair genes, K-ras, p53, E-cadherin, VEGF-C, podoplanin	Sedivy	(64)
Adenocarcinoma	Serum	Protein	ELISA	ng/ml	50			66.5 years (range 47–87)	mean ± std = 482 ± 170 ng/ml (range 192–919)				biomarker	p < 0.001		Koopmann	(42)
Healthy	Serum	Protein	ELISA	ng/ml	22			43.5 years (range	mean ± std = 204 ± 65 ng/ml (range								

								21–62)	69–370 ng/ml)								
Ductal adenocarcinoma	Tumor	Protein	IHC	Path score	73									correlation with MMP-9 and VEGF	66% smokers	Lazar	(66)
IPMN	Tumor	Protein	IHC	Path score	6										33% smokers		
Unresectable adenocacrinoma: galunisertib + gemcitabine	Plasma	Protein	multiplex assay										immune activation	Correlation to CD3+ epigenetic -0.21; Correlation to TGFβ 0.10		Melisi	(94)
Ductal adenocarcinoma	Tissue	mRNA	TCGA	Relative abundance										SPP1 expression and Treg abundance: rho = 0.283, p = 0.000129. SPP1 expression and macrophage abundance: rho = 0.272, p = 0.000237. M2: F = 32.74, p = 2.3 x 10E-8	enriched expression in macrophage and ductal cells	Chen	(95)
Pancreas cancer stage	Tissue	Protein	IHC	Allred score	11				Spearman correlation = 0.281				tumor progression	p = 0.403		Coppola	(74)
Stage I/II	Serum	Protein	ELISA	ng/ml	43				120±12 ng/ml							Kolb	(48)
Stage III/IV	Serum	Protein	ELISA	ng/ml	27				126±20 ng/ml								

Ductal adenocarcinoma	Tumor	Protein	IHC	% positivity	15				60%							Kolb	(48)
Lymph node and liver metastasis	Tumor	Protein	IHC	% positivity	11				72%								
Healthy pancreas	Tissue	Protein	IHC	% positivity	17				30%/5%						30% of acinar cells, 5% of ductal cells		
Ductal adenocarcinoma	Tumor	Protein	IHC	Positive/ negative	73				intensified staining, cell membrane and cytoplasm of tumor cells						MCP-1 colocalized in malignant ducts	Lazar	(67)
Lymph node metastasis	Tumor	Protein	IHC	Positive/ negative					intensely positive						MCP-1 intensely positive		
Healthy	Tumor	Protein	IHC	Positive/ negative					focally present, apical surface of ductal epithelium								
Ductal adenocarcinoma	Tumor	mRNA	RT-qPCR	Relative abundance	73									correlation between tissue OPN and MCP-1 mRNAs $p < 0.05$	66% smokers	Lazar	(67)
Premalignant lesion	Tumor	mRNA	RT-qPCR	Relative abundance	6										33% smokers		
ductal adenocarcinoma	Tumor	mRNA	RT-qPCR	Relative abundance	40				high=25, medium=8, low=5, none=2							Sullivan	(60)
IPMN	Tumor	mRNA	RT-qPCR	Relative abundance	6				high=0, medium=0, low=1, none=5								
Well-differentiated ductal adenocarcinoma	Tumor	Protein	IHC	Path score	15				183.8 for grade III					$p < 0.05$	grade; also correlation with LMX1A	Tsai	(43)

Moderately-differentiated ductal adenocarcinoma	Tumor	Protein	IHC	Path score	65				118.3 for grade II								
Poorly-differentiated Ductal adenocarcinoma	Tumor	Protein	IHC	Path score	20				87.1 for grade I						undetectable in healthy epithelia		
T4 ductal adenocarcinoma	Tumor	Protein	IHC	Path score	27				186.1					$p < 0.05$	stage T	Tsai	(43)
T3 ductal adenocarcinoma	Tumor	Protein	IHC	Path score	33				132.6								
T2 ductal adenocarcinoma	Tumor	Protein	IHC	Path score	27				99.5								
T1 ductal adenocarcinoma	Tumor	Protein	IHC	Path score	13				66.8								
N1 ductal adenocarcinoma	Tumor	Protein	IHC	Path score	55				148.3					$p < 0.05$	stage N	Tsai	(43)
N0 ductal adenocarcinoma	Tumor	Protein	IHC	Path score	45				98.8								
M1 ductal adenocarcinoma	Tumor	Protein	IHC	Path score	78				169.4					$p < 0.05$	stage M	Tsai	(43)
M0 ductal adenocarcinoma	Tumor	Protein	IHC	Path score	59.6				111.3								
Stage IV ductal adenocarcinoma	Tumor	Protein	IHC	Path score	37				169.4					$p < 0.05$	AJCC stage	Tsai	(43)
Stage III ductal adenocarcinoma	Tumor	Protein	IHC	Path score	13				143.8								
Stage II ductal adenocarcinoma	Tumor	Protein	IHC	Path score	30				106.2								
Stage I ductal adenocarcinoma	Tumor	Protein	IHC	Path score	20				70.6								
Ductal adenocarcinoma , stage 2	Serum	Protein	ELISA	ng/ml	10				~120					stage IV versus stage III ($P < 0.01$), stage III versus stage II ($P > 0.05$)		Rychlíková	(80)
Ductal adenocarcinoma , stage 3	Serum	Protein	ELISA	ng/ml	24				~100								

Ductal adenocarcinoma, stage 4	Serum	Protein	ELISA	ng/ml	29				~160								
Adenocarcinoma, early stage	Serum	Protein	Multiplex proximity ligation assay		96			65						not significant	significantly increased over IPMN P < 0.0001 (AUC 0.73, [0.65–0.80])	Song	(91)
Adenocarcinoma, late stage	Serum	Protein	Multiplex proximity ligation assay		92			64									
Cancer, stage I + II	Tissue	Protein	IHC	Path score	56				Tumor: 46 (82.1%) negative, 10 (17.9%) positive. Stroma: 20 (35.7%) negative, 36 (64.3%) positive					tumor: P = 0.642 stroma: P = 0.011		Cao	(65)
Cancer, stage III + IV	Tissue	Protein	IHC	Path score	19				Tumor: 14 (73.7%) negative; 5 (26.3%) positive. Stroma: 1 (5.3%) negative, 18 (94.7%) positive								
Cancer, stage T1 + T2	Tissue	Protein	IHC	Path score	15				Tumor: 13 (86.7%) negative, 2 (13.3%) positive. Stroma: 7 (46.7%) negative, 8 (53.3%) positive					tumor: P = 0.718 stroma: P = 0.139		Cao	(65)

Cancer, stage T3 + T4	Tissue	Protein	IHC	Path score	60				Tumor: 47 (78.3%) negative; 13 (21.7%) positive. Stroma: 14 (23.3%) negative, 46 (76.7) positive								
Cancer, stage N0	Tissue	Protein	IHC	Path score	52				Tumor: 44 (84.6%) negative, 8 (15.4%) positive. Stroma: 16 (30.8%) negative, 36 (69.2%) positive					tumor: P = 0.234 stroma: P = 0.422		Cao	(65)
Cancer, stage N1	Tissue	Protein	IHC	Path score	23				Tumor: 16 (69.6%) negative; 7 (30.4%) positive. Stroma: 5 (21.7%) negative, 18 (78.3%) positive								
Cancer, stage M0	Tissue	Protein	IHC	Path score	63				Tumor: 52 (82.5%) negative, 11 (17.5%) positive. Stroma: 21 (33.3%) negative, 42 (66.7%) positive					tumor: P = 0.386 stroma: P = 0.045		Cao	(65)

Cancer, stage M1	Tissue	Protein	IHC	Path score	12				Tumor: 8 (66.7%) negative; 4 (33.3%) positive. Stroma: 0 (0%) negative, 12 (100%) positive								
Ductal adenocarcinoma, by stage	Tissue	mRNA	TCGA	Relative abundance										Spearman: rho = 0.143, p = 0.0587	stages 1 - 4	Chen	(95)
High-grade IPMN	Tissue	mRNA	scRNASeq	Relative abundance	3											Li	(72)
IPMN-derived PDAC	Tissue	mRNA	scRNASeq	Relative abundance	2				higher expression								
Intraductal papillary mucinous neoplasm	Tissue	Protein	TCGA_PAAD	Path score	3				~ 2					p < 0.001		Li	(72)
Ductal adenocarcinoma	Tissue	Protein	TCGA_PAAD	Path score	2				~ 6								
Primary cancer	Tissue	Protein	Western blotting	Scan	8				~ 3					p < 0.001		Deng	(69)
Lymph node metastasis	Tissue	Protein	Western blotting	Scan	8				~ 17								
adjacent healthy tissue	Tissue	Protein	Western blotting	Scan	8				~ 1								
			Western blotting	Scan													
Lymph node metastasis	Tissue	Protein	Western blotting	Scan	8									OPN/RAN r = 0.9048, p < 0.01 ; OPN/AR r = 0.7549, p < 0.05		Deng	(69)
Ampullary cancer	Tumor	Protein	IHC	Path score	100				positive ITAM 36, positive MTAM 32, negative 32						positive TAMs associated with invasion, tumor stage, TNM stage, lymphovascular invasion, recurrence	Hsu	(63)

Ampullary cancer, non-OPN+ ITAMs	Tumor	Protein	IHC	Path score	39				51 months				survival			Hsu	(63)
Ampullary cancer, OPN+ ITAMs	Tumor	Protein	IHC	Path score	24				29 months								
Pancreatic cancer, OPN +	Tumor	Protein	IHC	Path score	93				17.1 months					p = 0.04		Collins	(45)
Pancreatic cancer, OPN -	Tumor	Protein	IHC	Path score	59				11.6 months								
Pancreatic cancer, OPN < 150 ng/ml	Serum	Protein	ELISA	ng/ml	79				337 days					95% CI, HR (269–435, 1.00) p = 0.007	p = 0.025 (multivariate analysis)	Poruk	(90)
Pancreatic cancer OPN > 150 ng/ml	Serum	Protein	ELISA	ng/ml	7				179 days					95% CI, HR (63 - ∞, 3.01)			
Locally advanced cancer	Serum	Protein	Multiplex proximity ligation assay		64									no association	36 biomarkers	Rao	(77)
Cancer, OPN high	Tissue	mRNA (TCGA)	Gene expression profile	GEPIA	45									overall: P = 0.0077 disease-free: P = 0.015		Cao	(65)
Cancer, OPN low	Tissue	mRNA (TCGA)	Gene expression profile	GEPIA	45												
Cancer, OPN high	Tissue	Protein	IHC	Path score	32				17.4 months					log rank p = 0.0858 Wilcoxon p = 0.0322	protective role of stromal OPN only in the initial years after surgery	Franklin	(47)
Cancer, OPN low	Tissue	Protein	IHC	Path score	35				10.3 months					Cox hazard ratio 0.8 (0.62–0.99), p = 0.046			
Cancer, OPN high	Tissue	mRNA												log rank p = 0.51 Wilcoxon p = 0.24		Franklin	(47)

Cancer, OPN low	Tissue	mRNA															
Ductal adenocarcinoma	Tumor, fine needle aspirate	mRNA	RT-qPCR	Relative abundance	40	17	23	68.1 (45–89)		579 days vs. 113 days, p=0.003	406 days vs. 749 days, p=0.049	530 days vs. 441 days, p=1		OPNa: HR 0.11 CI 0.01–1.06, p = 0.06. OPNb: HR3.0 CI 0.94–11.37, p = 0.06. OPNc: HR 1.13 CI 0.39–3.24, p = 0.74		Siddiqui	(73)
Sunitinib treatment overall survival	Serum	Protein	ELISA	ng/ml										coefficient = 0.0840 HR = 1.087 (1.011-1.169) p = 0.023	10 ng/ml	Jiménez-Fonseca	(93)
Sunitinib treatment progression-free survival	Serum	Protein	ELISA	ng/ml										coefficiant = 0.0606 HR = 1.062 (0.954-1.183) p = 0.269			
Ductal adenocarcinoma, long survival	Tissue	mRNA	RT-qPCR	Relative abundance	12				2	2	0	0		OPN p=0.031, OPNa p =0.6108, OPNb p = 0.0211, OPNc p = 0.0015	> 60 months post-surgery	Fiorino	(58)
Ductal adenocarcinoma, short survival	Tissue	mRNA	RT-qPCR	Relative abundance	9				6	3	4	6			< 24 months post-surgery		
Carcinoma, OPN high	Tissue	mRNA	gene expression profiling	Relative abundance	105									p = 0.0064	OncoLnc	Lu	(97)
Carcinoma, OPN low	Tissue	mRNA	gene expression profiling	Relative abundance	70												

Ductal adenocarcinoma	Tissue	mRNA	TCGA	Relative abundance	179									SPP1 low versus high p < 0.05		Chen	(95)
Postoperative ductal adenocarcinoma	Tissue	mRNA	RNASeq	Relative abundance	36										immune-related prognostic model: SPP1, LINC00683, SNHG10, LINC00237, and CASC19	Chen	(96)
Surrounding tissue	Tissue	mRNA	RNASeq	Relative abundance	36												
Ductal adenocarcinoma, OPN high	Tissue	RNA	TCGA_PAAD	Relative abundance										logrank p = 0.026		Li	(72)
Ductal adenocarcinoma, OPN low	Tissue	RNA	TCGA_PAAD	Relative abundance													
Ductal adenocarcinoma, OPN/macrophage high	Tissue	RNA	TCGA_PAAD	Relative abundance	70									logrank p = 0.0479		Li	(72)
Ductal adenocarcinoma, OPN/macrophage low	Tissue	RNA	TCGA_PAAD	Relative abundance	70												
Ductal adenocarcinoma, OPN high	Tissue	RNA	ICGC	Relative abundance	150									p = 0.0176 ; HR = 1.4596 (95% CI 1.0681-1.9946)		Li	(72)
Ductal adenocarcinoma, OPN low	Tissue	RNA	ICGC	Relative abundance	151												
Ductal adenocarcinoma, OPN high	Tissue	RNA	E-MTAB-6134	Relative abundance	144									p = 1 x 10-4 ; HR = 1.7973 (95% CI 1.338-2.4143)		Li	(72)

Ductal adenocarcinoma, OPN low	Tissue	RNA	E-MTAB-6134	Relative abundance	144												
Ductal adenocarcinoma, OPN high	Tissue	RNA	GSE-28735	Relative abundance	10									p = 0.0378 ; HR = 2.25323 (95% CI 1.047-4.8489)		Li	(72)
Ductal adenocarcinoma, OPN low	Tissue	RNA	GSE-28735	Relative abundance	32												
Ductal adenocarcinoma, OPN high	Tissue	RNA	GSE-71729	Relative abundance	31									p = 0.0453 ; HR = 1.6713 (95% CI 1.0108-2.7633)		Li	(72)
Ductal adenocarcinoma, OPN low	Tissue	RNA	GSE-71729	Relative abundance	94												
Ductal adenocarcinoma, OPN high	Tissue	RNA	TCGA	Relative abundance	45									overall: Logrank p=0.0077 ; HR(high)= 2.4 ; p(HR)=0.0096		Raymant	(96)
Ductal adenocarcinoma, OPN low	Tissue	RNA	TCGA	Relative abundance	45									disease-free: Logrank p=0.015 ; HR(high)= 2.3 ; p(HR)=0.017			
Pre-surgery	Serum	Protein	ELISA	ng/ml	22								cancer treatment			Kolb	(48)

Post-surgery	Serum	Protein	ELISA	ng/ml	22				>20% decrease 7/ 22 (32%) >20% increase 9/22 (41%) no change 6/22 (27%)								
Pancreatic cancer, pre-surgery	Plasma	Protein	ELISA	ng/ml	12				30.0 [10.8–77.2]					p = 0.002 vs healthy; p = 0.719 vs post-surgery	CEA correlated with OPN (r = 0.600, p = 0.039)	Franklin	(49)
Pancreatic cancer, post-surgery	Plasma	Protein	ELISA	ng/ml	12				28.7 [9.3–92.6]					p = 0.019 vs healthy	an increase of 10 ng/ml (postoperative) increased the hazard by 1.502 [95% CI 1.113–2.027]		
Non-malignant disease	Plasma	Protein	ELISA	ng/ml					11,6 [7,80-19,8]								
Hepatobiliary pancreatic cancer high surgical stress	Serum	Protein	ELISA	mg/dl	8				< 1 --> 15					p < 0.05	peak postoperative day 3	Sekiguchi	(92)
Hepatobiliary pancreatic cancer medium surgical stress	Serum	Protein	ELISA	mg/dl	54				< 1 --> 7					p < 0.05	peak postoperative day 3		
Hepatobiliary pancreatic cancer low surgical stress	Serum	Protein	ELISA	mg/dl	7				< 1 --> 2					n.s.			
Sunitinib responders 3 months	Serum	Protein	ELISA	ng/ml					28.9 ng/ml							Jiménez-Fonseca	(93)
Sunitinib non-responders 3 months	Serum	Protein	ELISA	ng/ml					40.9 ng/ml								

Sunitinib responders 6 months	Serum	Protein	ELISA	ng/ml					18.7 ng/ml					p = 0.039	6 months; baseline = 25.9 ng/ml		
Sunitinib non-responders 6 months	Serum	Protein	ELISA	ng/ml					38.5 ng/ml						6 months; baseline = 34.36 ng/ml		
Unresectable adenocacrinoma : galunisertib + gemcitabine	Plasma	Protein	Multiplex assay		104										changed during treatment: amphiregulin, CA15-3, cathepsin D, P-selectin, RAGE, sortilin, COMP, eotaxin-2, N-BNP, osteopontin, thrombospondin-4	Melisi	(94)
Unresectable adenocacrinoma : placebo + gemcitabine	Plasma	Protein	Multiplex assay		52												

[a]The reference citations pertain to the reference list in the main manuscript. Information is tabulated on those studies that investigated patients or patient material. The entries are arranged according to the cancer feature (or other disease feature) addressed, then by the underlying condition, then by year. N = number of subjects.

Table SIII. Cancer markers used in conjunction with Osteopontin.

Marker	Identity	Source	Function	Association	Outcome
CEA	Carcinoembryonic Antigen	Blood	Cancer marker	Strong correlation	
CA19-9	Cancer Antigen 19-9	Blood	Cancer marker	Strong correlation	
CA72-4	Cancer Antigen 72-4	Blood	Cancer marker	Strong correlation	
FOXM1	Forkhead Box M1	Tissue	Transcription factor	Correlation (protein, RNA)	Poor prognosis
LMX1A	LIM Homeobox Transcription Factor 1α	Tissue	Transcription factor	Correlation	Grade, stage
P53	Tumor Protein P53	Tissue	Transcription factor cell cycle regulator	No correlation (RNA)	
CDH1	E-Cadherin	Tissue	Adhesion molecule	No correlation	
AR	Androgen Receptor	Tissue	Surface receptor	Correlation	
MMP-9	Metalloproteinase-9	Tissue	Invasion	Correlation	
MCP-1	CCL-2	Tissue	Chemokine	Colocalization, correlation	
VEGF	Vascular Endothelial Growth Factor	Tissue	Angiogenesis	Correlation	
sTIE2	Soluble Angiopoietin-1 Receptor	Cystic fluid	Angiogenesis	Correlation	Benign cyst
RAN	Member RAS Oncogene Family	Tissue	Signal transduction small G protein	Correlation	(none identified)
CTNNB1	β-Catenin	Tissue	Signal transduction	No correlation	
KRAS	KRAS GTPase	Tissue	Signal transduction	No correlation (RNA)	
	bilirubin	Blood	Liver function	Correlation	
AST	Aspartate Transferase	Blood	Liver function	Correlation	
ALP	Alkaline Phosphatase	Blood	Liver function	Correlation	
GGT	Gamma-Glutamyl Transferase	Blood	Liver function	Correlation	
ALB	Albumin	Blood		Negative correlation	
LEP	Leptin	Cystic Fluid	Energy balance	Correlation	Benign cyst
	HDL cholesterol	Blood		Negative correlation	

Row-by-row, the table displays the marker, its identity, its function, the nature of its association with osteopontin, and the disease outcome it reflects. The shades are indicative of the strength of correlation or the associations with clinical outcomes.

Table SIV. Blood biomarker osteopontin.

Source	Cancer	IPMN	Pancreatitis	Obesity	Diabetes	Healthy
Plasma	2x					Baseline
Plasma	Elevated			Not a marker		Baseline
Plasma	Elevated		Reference		Reference	Baseline
Plasma	Elevated					Baseline
Serum	Elevated (part of 6-plex immunoassay)	Delineation	Delineation			Baseline
Serum	at 2x std: 80 sensitivity, 97% specificity					Baseline
Serum	(cancer vs healthy) 34% sensitivity, 94% specificity		(cancer vs. pancreatitis) 34% sensitivity, 92% specificity			Baseline
Serum	95% sensitivity, 100% specificity		Precipitous drop in sensitivity for cancer			Baseline
Serum	1.6-fold over healthy		1.9-fold over healthy			Baseline

Osteopontin (not depicting splice variants) in plasma or serum of adenocarcinoma patients, compared to non-cancerous conditions of the pancreas and to healthy controls.

Table SV. Osteopontin in pancreatic cancer immune responses.

Cell type	Characteristics	OPN	Comment
T-lymphocytes	Exhaustion	Mediator	Secreted from suppressor cells
	Recruitment	Mediator	Papillary mucinous neoplasm
B-lymphocytes	Recruitment	Mediator	Papillary mucinous neoplasm
Macrophages	Occasional peritumoral cells	Positive	
	Tumor-infiltrating macrophages	Strong	Healthy tissue/distant cells negative
	Mainly ductal cells	Positive	5-Gene signature, risk score formula
		Weak	Intraductal tubulary neoplasm
	Tumor-associated cells	Positive	Bulky ampullary neoplasm
	Reprogramming from M1 to tumor-associated macrophages	Mediator	recruited from blood or bone marrow
	M2 cells	Accumulation	Adenocarcinoma, OPN-CD44 signaling
Monocytic myeloid-derived suppressor cells	Immune escape	Mediator	
Osteoclast-like giant cells	No correlation with OPN in tumor cells	Positive	
Fibroblasts	Reprogrammed to cancer-associated fibroblasts	Mediator	Recruited from blood or bone marrow
	Myofibroblastic, metastasis-associated fibroblasts	Secreted	Immunosuppression

The table depicts a summary of published findings.

Table SVI. Osteopontin in diabetes.

Type	Susceptibility	Protection
Type 1	Autoantigen of the somatostatin cells	Regulation of early islet autoimmune damage
	Susceptibility by the a-type allele	Disease slowing
	Both Th1 and Th2 cytokines	
Type 2	Immunomediated Insulin resistance	Stimulated by glucose and incretins
	proinflammatory cytokine	Promotes cell metabolic activity
		Glucose-stimulated Insulin secretion
		Islet cell-cell connections
Healthy		Undifferentiated pancreatic precursors
		Epithelial-mesenchymal transitory fate decision
		Protective against cytotoxicity and hyperglycemia
		Facilitation of Insulin release
		Suppression of IL-1β-induced nitric oxide

In type 1 as well as type 2 diabetes, osteopontin may assume complex roles, which can either increase susceptibility or slow the disease progression. The bottom section depicts osteopontin functions in healthy pancreas.
